# Audiovisual Tracking of Multiple Speakers in Smart Spaces

**DOI:** 10.3390/s23156969

**Published:** 2023-08-05

**Authors:** Frank Sanabria-Macias, Marta Marron-Romera, Javier Macias-Guarasa

**Affiliations:** Universidad de Alcalá, Department of Electronics, Engineering School, Campus Universitario, 28805 Alcalá de Henares, Spain; frank.sanabria@edu.uah.es (F.S.-M.); marta.marron@uah.es (M.M.-R.)

**Keywords:** smart spaces, audiovisual tracking, speaker localization, particle filter, multi-pose face observation model, probabilistic SRP-PHAT

## Abstract

This paper presents GAVT, a highly accurate audiovisual 3D tracking system based on particle filters and a probabilistic framework, employing a single camera and a microphone array. Our first contribution is a complex visual appearance model that accurately locates the speaker’s mouth. It transforms a Viola & Jones face detector classifier kernel into a likelihood estimator, leveraging knowledge from multiple classifiers trained for different face poses. Additionally, we propose a mechanism to handle occlusions based on the new likelihood’s dispersion. The audio localization proposal utilizes a probabilistic steered response power, representing cross-correlation functions as Gaussian mixture models. Moreover, to prevent tracker interference, we introduce a novel mechanism for associating Gaussians with speakers. The evaluation is carried out using the AV16.3 and CAV3D databases for Single- and Multiple-Object Tracking tasks (SOT and MOT, respectively). GAVT significantly improves the localization performance over audio-only and video-only modalities, with up to 50.3% average relative improvement in 3D when compared with the video-only modality. When compared to the state of the art, our audiovisual system achieves up to 69.7% average relative improvement for the SOT and MOT tasks in the AV16.3 dataset (2D comparison), and up to 18.1% average relative improvement in the MOT task for the CAV3D dataset (3D comparison).

## 1. Introduction

Smart spaces are environments equipped with a set of monitoring sensors, communication, and computing systems. The primary objective of smart spaces is understanding people’s behavior within them and their interactions, to improve human–machine interfaces. In this context, one of the required core low-level information is the presence, position, orientation (pose), and voice activity of users within the space, as these features play a significant role in high-level behavior and interaction understanding between the users and the environment.

Many of the approaches proposed in the literature for people localization within a monitored scene use information from a single type of sensor (video cameras [1,2,3], microphone arrays [4,5,6], infrared beacons [7,8,9], and others). Among them, the most used ones are video cameras and microphone arrays. In applications such as “smart” video conferencing, human–machine interfacing [10], automatic scene analysis [11], automatic camera tracking [12], and far-field speech recognition [13] it would be possible to locate and monitor speakers or audio sources within the space using the audio or video information. Additionally, providing accurate speaker positions could facilitate other tasks such as speech recognition [14].

Smart spaces are usually closed environments where the reverberation phenomenon is present, complicating the localization task [15] by audio means. Microphones are usually located in fixed positions on walls or ceilings. Therefore, the distance between the speech sources and the available microphones will lower the signal-to-noise ratio, making it difficult to locate the source [16]. Usually, video cameras are included in this kind of application to increase tracking accuracy as they provide additional information.

Nevertheless, video camera-only-based tracking systems also have their shortcomings. The uncertainties inherent to the acquisition system, the lighting conditions (brightness, shadows, contrast), and noise (sensor or optics characteristics) [17] decrease their accuracy in people pose extraction in real scenarios. Another problem in the vision-based systems is the total or partial occlusion of the subjects to be tracked [18] due to the light’s directional nature. In this context, audio signals are not strictly directional, especially at low frequencies. Hence, they are the perfect complement to the visual tracking process when targets are occluded or out of the camera’s field of view (FoV).

From the discussion above, it is clear that audio and video sources provide complementary information for people tracking in smart spaces. A combination of the best features from both sensors can thus improve the accuracy and robustness of the pursuit tracking process. This combined use of audio and video information in the tracking task is referred to as audiovisual tracking, in which the mouth is the element to track, as this is the area of maximum radiated acoustic power when speaking.

## 2. Previous Works

Given the increased availability of easy-to-deploy audio and video sensors and the improvements in computing facilities, in recent years, there has been a relevant growth in the number of proposals for multiple speakers tracking (Multiple-Object Tracking, MOT) in smart spaces, combining audio and video information [17,19,20,21,22,23,24,25,26,27,28,29,30,31,32]. Qian et al. in [17] conducted an extensive review of state of the art in audiovisual speaker tracking. In their review, different literature proposals were classified according to various aspects like the space used to perform the tracking (image plane, ground-level or three-dimensional—3D), the configuration of the sensor location (co-located or distributed), the number of sensors, the tracking method, among others. This review highlighted the main research lines and open problems in the audiovisual speaker tracking field.

Along the review, one of the most relevant research lines focuses on tracking a variable and an unknown number of speakers [19,27,33], as this is a still not fully solved problem today. Another line focuses on finding a better visual appearance model to track multiple speakers in indoor environments [34]. At the same time, other proposals are centered on audiovisual tracking in compact configurations (co-located camera and microphone array) for applications such as human-robot interaction [17,20,21,35].

In any case, a clear tendency in the literature proposals is the predominance of probabilistic tracking models such as Bayesian Filters (BFs, i.e., mainly Kalman filters and particle filters) [17,20,29,31,32,34,36,37]. The reasoning behind the use of BFs is that they provide a natural and robust framework to combine different sources of information, in this case, diverse sensing systems.

The reduction of the number of sensors is another important tendency in the literature. In recent proposals, most of the approaches address the tracking task only with only one camera, and one microphone array [17,20,27]. Many of these proposed systems track speakers in the image plane (2D) [32,38]. However, proposals addressing the localization in the full 3D space can also be found in [17,20,24,27,29,31], thus imposing additional challenges analyzed below.

Finally, in the MOT context, it is always necessary to handle situations, like targets occlusions or closely located, where care must be taken in the association between measurements and targets [20]. These situations could be very challenging when audio or visual observations fail or give low-confidence likelihood, thus presenting an interesting set of proposals in the literature, as analyzed in the following sections.

Another general aspect is the recent rise of deep learning techniques in audiovisual speaker tracking. The most commonly used approach is based on an observation model that keeps the Bayesian scheme. There are many examples of face detectors based on deep learning [25,26,27,28,29,31], although Siamese networks have also been used to generate measures of particle similarity to previous reference images of each target [32,39], and fusion models based on the attention mechanism [32]. Fewer proposals we found with end-to-end trained audiovisual solutions as in [24,40] for object tracking in which visual and auditory inputs are fused by an added fusion layer.

This paper presents a complete contribution to robust audiovisual multiple-speaker 3D tracking in smart spaces. The proposed approach is thoroughly analyzed and compared using the best available datasets published for this purpose. We also compare our method against, to the best of our knowledge, the best proposals in the state-of-the-art, emphasizing our focus on signal recognition and pattern analysis. This distinguishes our work from recent proposals that primarily rely on deep learning techniques.

### 2.1. Visual Tracking

An important issue to analyze within the visual part of the tracking is how to locate the mouth position in the image plane. Within a smart space, speakers could be far from sensors, at distances up to several meters, where detecting the mouth is challenging, as its size can be just a few pixels on the image. Given its small size, usually, the face is first located, as it is easier to detect, and then an estimation of the mouth location within the face area is made. This strategy is used in several proposals [17] and gives a chance to facial feature estimation with pattern recognition methods.

Typically, audiovisual speaker tracking literature uses color histogram-based likelihood estimations to locate faces in images [19,26,41]. However, this technique has proved to be not very accurate [17,32]. More accurate techniques for face location within the image are based on the use of trained detectors like the classic Viola and Jones (VJ) [42], or more recent ones based on deep learning [17].

Regarding the estimation of the mouth position within the face area, most face detectors used in audiovisual tracking are independent of the face pose, as in [17,20]. This approach has limited accuracy for mouth position estimation, as it depends on the face pose relative to the camera. To partially overcome this issue, in [17,20] the mouth localization is solved considering the aspect ratio of the face detection bounding box (BB).

Location in a 3D space using a single camera poses an additional challenge in the visual tracking part, as it requires estimating the target depth based on the scene projection on the 2D plane. From the 2D mouth position estimation, Qian et al. in [20] proposed a 3D projection assuming the shoulders width in 3D as a known parameter. In [17], as faces are detected in 2D with the face detection MXNet [43] network, the 3D mouth position is derived assuming that the face detection BB is related to the distance between the target and the camera.

A third issue with the mouth localization is its integration into the probabilistic tracking framework. In some works, like [17], when a face is located in 2D, a BB is projected to the 3D space, assuming a predefined probability distribution around its 3D points. As a more realistic proposal, in [44] a likelihood function for the face location is proposed, built with the internal information extracted from a modified VJ detector.

Most proposals [19,20,33] were evaluated in the AV16.3 database [45] where people’s faces were always visible on some of the available cameras (people do not turn their backs to all the cameras simultaneously). In [17], the more complex evaluation dataset CAV3D was presented, using a co-located audiovisual sensor setup, where faces are not always visible (sometimes people turn their backs to the camera), including strange poses and varied dynamic behaviors. This new condition implies that a face detector will not provide localization information as long as the camera does not see the faces, and thus another mechanism is required to allow the localization task to provide accurate estimations, generating another relevant challenge to be solved in the visual tracking task.

Some solutions implement a generative color histogram-based algorithm when the faces are not detected [17]. However, when complex contexts arise, with part of the target’s movement happening outside of the camera’s FoV (such as in CAV3D), the audio modality is the only one available, and no color-based approach can be used.

### 2.2. Audio Tracking

In audio localization, the most common approach is to compute the Generalized Cross-Correlation function (GCC) between pairs of microphones to generate an acoustic activation map with Steered Response Power (SRP) strategies, usually combined with the PHAT transform [20,46,47,48,49,50,51,52,53,54].The audio likelihood model thus obtained is then associated with the SRP acoustic map. The spatial resolution of these methods strongly depends on the array geometry, and for small microphone arrays (short distance between microphones, compared to the search space area), SRP presents a wide active response (low resolution), mainly in radial distance from the source [17].

Another problem in audio localization is room reverberation, which generates multiple peaks in GCC. To alleviate this effect, the approach in [55] proposed to model GCC as a Gaussian mixture. This strategy allows associating only one GCC Gaussian (or peak) with the source.

For multiple-speaker tracking (MOT), the association between GCC peaks and speakers must be addressed, taking also into account that when more than one speaker is in the same direction relative to the microphone array position, the GCC peaks related to the different speakers are mixed. To address this problem, ref. [55] proposes to use an a priori distribution from a Kalman filter to assign each peak to one of the speakers in each pair of microphones, where the assignment is made independently for each pair of microphones.

In [29], the authors propose a phase-aware VoiceFilter and a separation before the localization method. They separate the speech from different speakers by first using VoiceFilter with phase estimation and then applying a localization algorithm. However, the method needs clean speech samples for each speaker in the training phase, thus limiting its applicability. In [32], a novel acoustic map based on a spatial–temporal Global Coherence Field (stGCF) map is proposed, which utilizes a camera model to establish a mapping relationship between the audio and video localization spaces.

### 2.3. Contributions

The main contributions of our proposal (named GAVT) are: (i) providing an audiovisual localization approach in which the visual strategy is based on exploiting the knowledge of a trained face detector, modified to generate a likelihood model. This approach will thus not depend on a standard face detection process; (ii) using a pose-dependent strategy that improves the 2D mouth location estimation, thanks to the likelihood model extension with a new in-plane rotation and an image evaluation exploration; (iii) proposing a mechanism to handle MOT tasks for visual observations dependent on the new likelihood dispersion; and finally (iv) presenting a novel mechanism to avoid target interference in audio tracking that selects the more adequate GCC peak for each target, based on the joint distribution of all pairs of microphones.

The evaluation will be done on the AV16.3 and CAV3D datasets, both in single and multiple-speaker scenarios, to provide realistic and comparative quantitative and qualitative results and contributions within all the challenges exposed in this state-of-the-art revision.

The remainder of this paper is structured as follows: Section 3 describes the notation used and the definition of the multi-speaker tracking problem. Section 4 describes the general scheme of our proposal, while Section 5 and Section 6 detail the proposed video and audio observation models, respectively. The experimental setup and results obtained are described correspondingly in Section 7 and Section 8. Finally, Section 9 presents the paper conclusions.

## 3. Notation and Problem Statement

Real scalar values are represented by lowercase letters (e.g., α,c). Vectors are represented by lowercase bold letters (e.g., x). Matrices represented by uppercase bold letters (e.g., M). Uppercase letters are reserved to define vector and set sizes (e.g., vector $y={\left(y_{1},\cdots ,y_{M}\right)}^{T}$ is of size *M*). Calligraphic fonts are reserved to represent sets (e.g., R for real or G for generic sets). The $l_{p}$ norm (p>0) of a vector is depicted as ${∥.∥}_{p}$, e.g., ${∥x∥}_{p}={\left(|x_{1}{|}^{p}+\cdots +{\left|x_{N}\right|}^{p}\right)}^{1/p}$, where |.| is reserved to represent absolute values of scalars or the module operation for complex values. The $l_{2}$ norm ${∥.∥}_{2}$ (Euclidean distance) will be written by default as ∥.∥ for simplicity. The discrete Fourier transform of a discrete signal x[n] is represented with the complex function X[ω], with $X^{*}\left[\omega \right]$ being the complex-conjugate of X[ω]. Along the paper, we will use the ${}^{a}$, ${}^{v}$, and ${}^{av}$ superscripts to refer to elements belonging to the audio, video, and audiovisual modalities, respectively. Moreover, the tilde (^~^) in $\tilde{X}$ refers to the projection of *X* in a different space.

Within the notation context defined above, let us consider an indoor environment with a set of $N_{M}$ microphones $M=\{m_{1},m_{2},\cdots ,m_{N_{M}}\}$, where $m_{\nu }$ is a known three-dimensional vector $m_{\nu }={\left(m_{\nu x},m_{\nu y},m_{\nu z}\right)}^{T}$ denoting the position of the $\nu {}^{th}$ microphone from the reference coordinate origin. For processing purposes, the microphones are grouped in pairs, as elements in a set $Q=\{\pi {}_{1},\pi {}_{2},\cdots ,\pi {}_{N_{Q}}\}$, where $\pi {}_{j}=\left(m_{j_{1}},m_{j_{2}}\right)$ is composed of two three-dimensional vectors $m_{j_{1}}$ and $m_{j_{2}}$, ($m_{j_{1}},m_{j_{2}}\in M$, with $m_{j_{1}}\neq m_{j_{2}}$) that describe the spatial location of the microphones in the pair *j*. If all microphone pairs are allowed, then $N_{Q}=N_{M}\cdot \left(N_{M}-1\right)/2$.

Given this setup, let us assume that there is a set of $N_{S}$ acoustic sources $S=\{r_{1},r_{2},\cdots ,r_{N_{S}}\}$, where $r_{i}$ is a known three-dimensional vector $r_{i}={\left(r_{ix},r_{iy},r_{iz}\right)}^{T}$, emitting $N_{S}$ acoustic signals $x_{i}\left(t\right)$, which are received by each microphone $m_{\nu }$ obtaining $N_{M}$ time signal $s_{\nu }\left(t\right)$ according to the propagation model in Equation (1):

(1) $$s_{\nu }\left(t\right)=\sum_{i=1}^{N_{S}}\left[h_{i,\nu }\left(t\right)*x_{i}\left(t\right)+\eta_{i,\nu }\left(t\right)\right],$$

with $h_{i,\nu }\left(t\right)$ being the Room Impulse Response (RIR) between the acoustic source position $r_{i}$ and the $\nu {}^{th}$ microphone, * the convolution operator, and $\eta_{i,\nu }\left(t\right)$ a signal that models all the audio signal adverse effects not included in $h_{i,\nu }\left(t\right)$ (noise, interference, etc.).

In an anechoic (free-field) condition the signals received by each microphone are just a delayed and attenuated version of the acoustic source signal, as shown in Equation (2):

(2) $$s_{\nu }\left(t\right)=\sum_{i=1}^{N_{S}}\left[\alpha_{i,\nu }\cdot x\left(t-\tau_{i,\nu }\left(r_{i}\right)\right)\right],$$

where $\tau_{i,\nu }\left(r_{i}\right)=c^{-1}∥r_{i}-m_{\nu }∥$ is the propagation delay between $m_{\nu }$ and $r_{i}$, $\tau_{i,\nu }\left(r_{i}\right)$, $\alpha_{i,\nu }=\frac{1}{4\pi c\tau_{i,\nu }\left(r_{i}\right)}$ is a distance-related attenuation assuming spherical propagation [56], and *c* is the sound propagation velocity in air.

The environment is also equipped with a set of $N_{C}$ cameras $C=\{c_{1},c_{2},\cdots ,c_{N_{C}}\}$, giving the corresponding $I_{\theta }\left(t\right)$ images, where $c_{\theta }$ is a known three-dimensional vector $c_{\theta }={\left(c_{\theta x},c_{\theta y},c_{\theta z}\right)}^{T}$, denoting the position of the $\theta {}^{th}$ camera from the reference coordinate origin. The $K_{\theta }$ intrinsic calibration matrix for each camera is also available, with $\theta =1\ldots N_{C}$.

In the geometrical discussions, we will refer to the 3D space as that in which every point is defined by its 3D coordinates ${\left(x,y,z\right)}^{T}$. The visual observation space will be defined in the 2D plane (image plane) as ${\left(u,v,s\right)}^{T}$, where (u,v) are the pixel coordinates in the image plane, and *s* refers to the *size* of the explored image plane.

The proposal in this work will be exploiting the formulation of Bayesian Filters (BFs), which are techniques that estimate the posterior probability density function (PDF) of a system state whose dynamics are statistically modeled along time, given a set of observations or measurements [57], also statistically modeled.

In these models, the state ($x{}_{n}$) and observation ($z{}_{n}$) vectors can be mathematically obtained along time, being n the corresponding time instant. The state vector $x{}_{n}$ characterizes the properties of the system to be estimated (e.g., position, velocity, physical dimensions), and the observation vector $z{}_{n}$ considers the measurements from the system behavior with all sensors in the environment.

In the BF framework, the estimation is made in two steps: prediction and update (also referred to as correction [57]). In the prediction stage, a prior PDF of the state vector is computed given its previous value $p(x{}_{n-1}|Z{}_{n-1})$ and using the state model $p(x{}_{n}|x{}_{n-1})$, as shown in Equation (3). In the update stage, a new posterior PDF is computed $p(x{}_{n}|Z{}_{n})$ (see Equation (4)) from the prior obtained in the prediction stage, and by including there the current observation vector information $z{}_{n}$, through the observation model $p(z{}_{n}|x{}_{n}$) and its likelihood $p(z{}_{n}|Z{}_{n-1}$).

(3) $$p\left(x{}_{n}|Z{}_{n-1}\right)=\int p\left(x{}_{n}|x{}_{n-1}\right)p\left(x{}_{n-1}|Z{}_{n-1}\right)dx{}_{n-1}$$

(4) $$p\left(x{}_{n}|Z{}_{n}\right)=\frac{p\left(z{}_{n}|x{}_{n}\right)\cdot p\left(x{}_{n}|Z{}_{n-1}\right)}{p(z{}_{n}|Z{}_{n-1})},$$

where $Z{}_{n}=\left\{z{}_{1},z{}_{2},\cdots ,z{}_{n}\right\}$.

Particle filters (PFs) are a particular class of BFs that approximate the state distribution with a set of weighted samples $\{x{}_{n}^{i},w_{n}^{i}\;$/$i=1,\cdots ,N_{P}\}$, called particles, that characterize each estimation hypothesis, as shown in Equation (5):

(5) $$p\left(x{}_{n}|Z{}_{n}\right)\approx \sum_{i=1}^{N_{P}}w_{n}^{i}\cdot \delta \left(x{}_{n}-x{}_{n}^{i}\right),$$

where $N_{P}$ is the number of particles, and $w_{n}^{i}$ are the weights characterizing the probability of every given particle $x{}_{n}$ or state value hypothesis.

The update stage is then carried out by applying *Importance Sampling* [57] (IS, and its derivation *Sequential Importance Resampling*, SIR), which is a statistical technique to estimate the properties of a posterior distribution $p(x{}_{n}|Z{}_{n})$, when its samples are generated from another sampled one, the a prior PDF in this case $p(x{}_{n}|Z{}_{n-1})$.

In this work, PFs are used for tracking the mouth position of multiple speakers in a smart space using audio and video information, so that the observation vector $z{}_{n}$ will be composed of observations from the audio modality $z{}_{n}^{a}$ and from the video modality $z{}_{n}^{v}$, so that $z{}_{n}={\left(z{}_{n}^{a},z{}_{n}^{v}\right)}^{T}$.

## 4. Audiovisual Tracking: The GAVT System Proposal

The tracking task will be thus carried out using only one video camera and one small microphone array in both co-located and distributed scenarios. It is assumed that the sensors are calibrated, and the audio and video signals are synchronized. It is also considered that there are a constant (and known) number of speakers (targets) in the space and that speakers do not stop talking for long periods. The problem complexity is thus concentrated in the multimodal 3D localization and tracking task in a multiple-speaker context, within a probabilistic approach without a reidentification process to solve the possible association problems that may appear in such context. Within this framework, and following the notation and the problem statement previously described, in this section we are presenting the global system proposed for addressing the audiovisual tracking task.

### 4.1. General Architecture

In our proposal, each target speaker or objective o, will be characterized at any time instant *n* by a state vector $x_{n,o}={\left(p_{n,o},{\dot{p}}_{n,o}\right)}^{T}$, where $p_{n,o}={\left(x_{n,o},y_{n,o},z_{n,o}\right)}^{T}$ is the speaker 3D position, and ${\dot{p}}_{n,o}={\left({\dot{x}}_{n,o},{\dot{y}}_{n,o},{\dot{z}}_{n,o}\right)}^{T}$ its velocity.

A separated particle filter is used for tracking each target o, with the SIR algorithm, using a set of $N_{P}$ particles ($N_{P}$ is assumed to be fixed for all targets and time instants). Each particle *i* ($i=1\ldots N_{P}$) at any time instant *n* will be characterized by a state vector $x_{n,o}^{i}={\left(p_{n,o}^{i},{\dot{p}}_{n,o}^{i}\right)}^{T}$, where $p_{n,o}^{i}={\left(x_{n,o}^{i},y_{n,o}^{i},z_{n,o}^{i}\right)}^{T}$ is the particle 3D position, and ${\dot{p}}_{n,o}^{i}={\left({\dot{x}}_{n,o}^{i},{\dot{y}}_{n,o}^{i},{\dot{z}}_{n,o}^{i}\right)}^{T}$ its velocity. Therefore, the set of $N_{P}$ particles will be $P{}_{n,o}=\left\{x_{n,o}^{1},x_{n,o}^{2},\cdots x_{n,o}^{N_{P}}\right\}$.

Following the standard PF framework, each particle set for a target o from a previous time instant $P{}_{n-1,o}=\left\{x{}_{n-1}^{1}{}_{,o},x{}_{n-1}^{2}{}_{,o},\cdots {x{}_{n-1}^{N_{P}}}_{,o}\right\}$, will be propagated according its corresponding state model $p(x_{n,o}|x{}_{n-1}{}_{,o})$, giving the predicted particle set $P{}_{n|n-1,o}=\left\{x{}_{n}^{1}{}_{|n-1,o},x{}_{n}^{2}{}_{|n-1,o},\cdots {x{}_{n}^{N_{P}}}_{|n-1,o}\right\}$, on a frame by frame basis. This set represents the sampled version of the prior distribution $p(x_{n,o}|Z{}_{n-1}{}_{,o})$ for each tracked target *o*, and thus completes the prediction stage of the standard BF framework in Equation (3).

Then, during the update stage at time *n*, audio and video data $z{}_{n}^{av}={\left(z{}_{n}^{a},z{}_{n}^{v}\right)}^{T}$ are used to evaluate, for every target *o* and predicted particle *i*, the *audiovisual* likelihood $l^{av}\left(p_{n,o}^{i}\right)$, combining the likelihoods calculated from the audio and video modalities, $l^{a}\left(p_{n,o}^{i}\right)$ and $l^{v}\left(p_{n,o}^{i}\right)$, respectively, so that $l^{av}\left(p_{n,o}^{i}\right)=f\left(l^{a}\left(p_{n,o}^{i}\right),l^{v}\left(p_{n,o}^{i}\right)\right)$.

Likelihood calculations are carried out for each predicted particle using the corresponding target observation model $p(z{}_{n}{}_{,o}|x_{n,o})$.

For every o target, the particles’ weights $\left\{w_{n,o}^{1},w_{n,o}^{2},\cdots w_{n,o}^{N_{P}}\right\}$ are obtained, conforming the sampled version of the likelihood $p(z{}_{n}{}_{,o}|Z{}_{n-1}{}_{,o})$ at the PF standard framework correction step in Equation (4).

After the update process, the particle set is resampled with the multinomial resampling method proposed in [58]. This way, particles with low weights are eliminated and those with high weight are replicated, keeping constant the number of particles $N_{P}$ used to characterize the state hypothesis of each estimation of the speaker o position at that time instant.

As a final result, we obtain the new particle set $P{}_{n,o}=\left\{x_{n,o}^{1},x_{n,o}^{2},\cdots x_{n,o}^{N_{P}}\right\}$, that constitutes the sampled version of the posterior PDF $p(x_{n,o}|Z{}_{n}{}_{,o})$. This final new particle set will be the one used as the prior distribution in the next time step.

**Figure 1:**
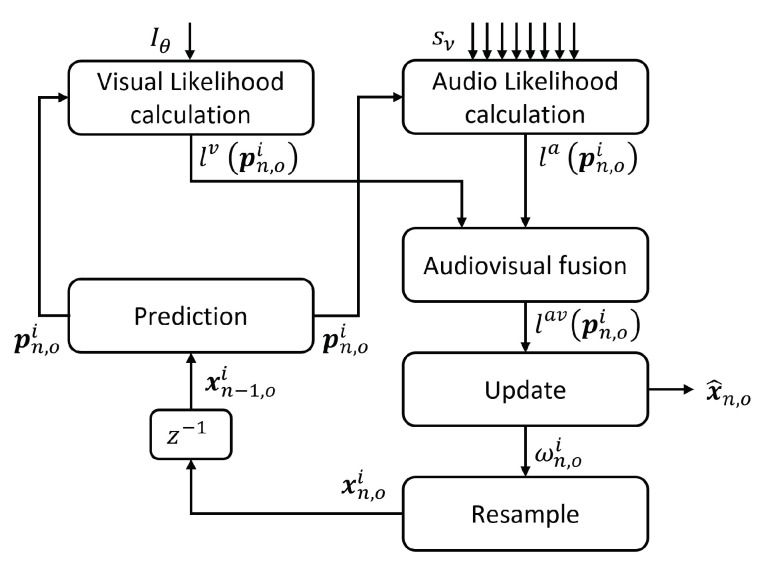
Particle filter general scheme for multiple speakers audiovisual tracking.

The general scheme described above is graphically presented in Figure 1.

### 4.2. Prediction

The state model used at each iteration of the PF to propagate the particles is the Langevin motion model [59], commonly used in the acoustic speaker tracking literature [60], as shown in Equation (6):

(6) $$x_{n,o}^{i}=Ax_{n-1,o}^{i}+Qu,$$

where A and Q are the transition and noise state matrices, respectively, u∼N(0,Σ) is the noise related to the process or state, for which a normal distribution is assumed, with zero mean 0 and Σ covariance matrix.

Matrix A corresponds to a first-order motion behavior, and it is described in Equation (7):

(7) $$A=\left[\begin{array}{cccccc} 1 & 0 & 0 & \;\epsilon \Delta_{T} & \;0 & \;0\\ 0 & 1 & 0 & \;0 & \;\epsilon \Delta_{T} & \;0\\ 0 & 0 & 1 & \;0 & \;0 & \;\epsilon \Delta_{T}\\ 0 & 0 & 0 & \;\epsilon & \;0 & \;0\\ 0 & 0 & 0 & \;0 & \;\epsilon & \;0\\ 0 & 0 & 0 & \;0 & \;0 & \;\epsilon \end{array}\right],$$

Moreover, the coefficients of Q matrix are given by Equation (8):

(8) $$Q=diag(\zeta \Delta_{T},\zeta \Delta_{T},\zeta \Delta_{T},\zeta ,\zeta ,\zeta ),$$

where $\Delta_{T}$ is the time interval (in seconds) between frames n and n−1, $\epsilon =e^{-\beta \Delta_{T}}$ and $\zeta =\bar{v}\sqrt{1-\epsilon^{2}}$ are the process’ noise model parameters, and diag(·) is a diagonal matrix with the diagonal values being its arguments. The control parameters used in the proposal are a steady-state velocity $\bar{v}$ and its velocity rate of change β, following the formulation in [59].

### 4.3. Update and Position Estimation

To fuse the audiovisual information generated from the audio and video sources, we assume independence between these modalities, so that they fulfill Equation (9):

(9) $$p\left(z{}_{n,o}^{av}|x_{n,o}\right)=p\left(z{}_{n,o}^{a}|x_{n,o}\right)\cdot p\left(z{}_{n,o}^{a}|x_{n,o}\right)$$

In practice, this means that the audiovisual likelihoods are obtained by computing the product of the likelihoods from both modalities, as shown in Equation (10):

(10) $$p\left(z{}_{n,o}^{av}|x_{n,o}\right)∼l^{av}\left(p_{n,o}^{i}\right)=f\left(l^{a}\left(p_{n,o}^{i}\right),l^{v}\left(p_{n,o}^{i}\right)\right)=l^{a}\left(p_{n,o}^{i}\right)\cdot l^{v}\left(p_{n,o}^{i}\right)$$

Then, following the sampled background of PFs presented in Equation (5), weights are updated by their likelihood values as shown in Equation (11):

(11) $$w_{n,o}^{i}=l^{av}\left(p_{n,o}^{i}\right)$$

Finally, the most probable state for each o target (${\hat{x}}_{n,o}$), thus the deterministic instantaneous value for $p(x_{n,o}|Z{}_{n}{}_{,o})$, is estimated evaluating Equation (12) [58].

(12) $${\hat{x}}_{n,o}=\sum_{i=1}^{N_{P}}w_{n,o}^{i}\cdot x_{n,o}^{i}$$

## 5. Video Observation Model

**Figure 2:**
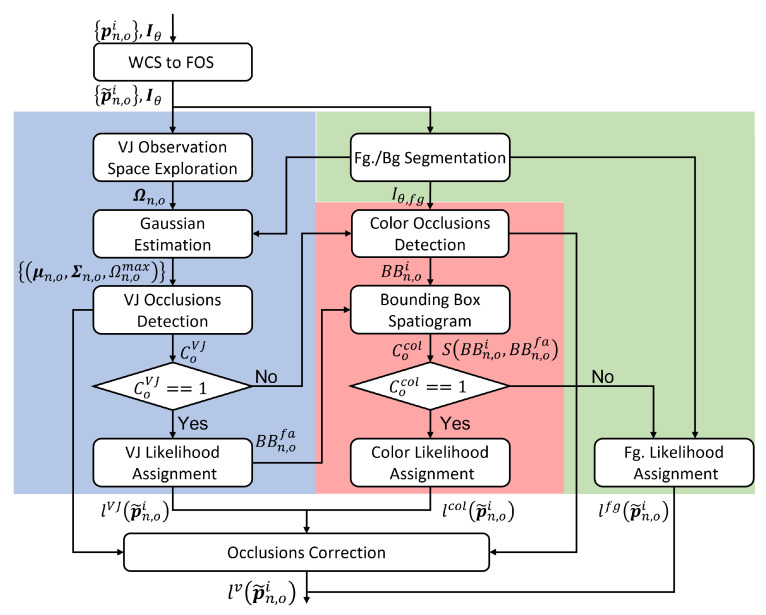
Video observation model: VJ Likelihood (light blue) Color-based Likelihood (light red) and Fg. Likelihood (light green) blocks.

The visual likelihood $l^{v}\left({\tilde{p}}_{n,o}^{i}\right)$ of the video observation model in the real 3D coordinate system, consists of three blocks, as shown in Figure 2, and explained below:

- The first block is an appearance-based observation model based on the VJ likelihood $l^{VJ}\left({\tilde{p}}_{n,o}^{i}\right)$. Using the probabilistic version of the VJ detector, this appearance-based algorithm accurately estimates the face and mouth location, taking into account different face poses, and generating a likelihood $l^{VJ}\left({\tilde{p}}_{n,o}^{i}\right)$ to them, as no tracking-by-detection is performed.
- The second block is based on a color histogram (color-based likelihood) $l^{col}\left({\tilde{p}}_{n,o}^{i}\right)$: The color histogram model is intended to be used when the first block fails to detect the face. These cases may appear with poses not correctly handled by the VJ model, for example, with the head tilted too far forward, or due to a person’s rapid movement, which blurs facial features and may cause a poor response from the VJ model.
- The third block is a foreground versus background segmentation (Fg./Bg. Segmentation) that generates a foreground likelihood (Fg. Likelihood) $l^{fg}\left({\tilde{p}}_{n,o}^{i}\right)$, which is used to validate the proposals from both previous models and restrict the video observation hypothesis dispersion, providing a $l^{fg}\left({\tilde{p}}_{n,o}^{i}\right)$ likelihood, when the other two components fail.

Thus, the general processing sequence of the video observation model $l^{v}\left({\tilde{p}}_{n,o}^{i}\right)$, shown in Figure 2, is as follows:

- The first step is to apply the coordinate transformation from the World Cartesian coordinates System (WCS) to the Face Observation Space (FOS) one. There, the tilde in ${\tilde{p}}_{n,o}^{i}$ refers to the projection of $p_{n,o}^{i}$ in this FOS. Thus, after projection, the algorithm determines which observation hypotheses are in the camera’s FoV.
- Then, foreground versus background segmentation (Fg./Bg. Segmentation) is applied. For each target o, the FOS is explored with VJ, looking for face detection, so in positive case $C_{o}^{VJ}=1$.
- For targets within the FoV that have no observation response from the VJ model (i.e., $C_{o}^{VJ}=0$), the color-based model (light red box in Figure 2) is applied if possible. If this is the case, $C_{o}^{col}=1$.
- If neither the VJ nor the color models can be applied, the foreground likelihood will be assigned.
- In any case, a mechanism to prevent a specific person’s visual hypothesis from being confused with observations from another target, an occlusion detector is included (in the VJ and Color modules). Thus, a procedure to restrict the likelihood analysis from hypotheses declared as occluded (Occlusions Correction block) is applied.

Following the observational model, a global visual likelihood function or video observation model $l^{v}\left({\tilde{p}}_{n,o}^{i}\right)$ is finally defined as in Equation (13), where a confidence level is assigned according to each model component.

(13) $$p\left(z{}_{n,o}^{v}|x_{n,o}^{i}\right)∼l^{v}\left({\tilde{p}}_{n,o}^{i}\right)=\left\{\begin{array}{cc} l^{VJ}\left({\tilde{p}}_{n,o}^{i}\right) & ifC_{o}^{VJ}\\ l^{col}\left({\tilde{p}}_{n,o}^{i}\right) & ifC_{o}^{VJ}=0\;\mathrm{and}\;C_{o}^{col}=1\\ l^{fg}\left({\tilde{p}}_{n,o}^{i}\right) & otherwise \end{array}\right.$$

If the measurement found with the appearance model $p(z{}_{n,o}^{v}|x_{n,o}^{i})$ is reliable, $C_{o}^{VJ}=1$ and the likelihood of related hypotheses are weighted according to the similarity Gaussian function $l^{VJ}\left({\tilde{p}}_{n,o}^{i}\right)$. If the VJ estimation is not reliable but the color model does show significant similarity to the reference model ($C_{o}^{col}=1$), the related hypotheses are weighted according to their similarity to this reference model as $l^{col}\left({\tilde{p}}_{n,o}^{i}\right)$. Finally, if both models do not provide confident enough measurements, the hypotheses are weighted according to the background subtraction model $l^{fg}\left({\tilde{p}}_{n,o}^{i}\right)$.

In the following subsections, the different blocks of the video observation model are further described and analyzed.

### 5.1. From World Coordinate System to Face Observation Space (WCS to FOS)

The person (its mouth) position hypotheses in the WCS $\left\{p_{n,o}^{i}\right\}=\left\{{\left(x_{n,o}^{i},y_{n,o}^{i},z_{n,o}^{i}\right)}^{T}\right\}$ are projected to the camera image plane using the pin-hole model. Thus, a collection of 2D points $\left(u_{n,o}^{i},v_{n,o}^{i}\right)$ and distances to the camera $d_{n,o}^{i}$ are obtained, to be validated through the video observation model.

Therefore, these 2D points represent hypotheses of the speakers’ mouth position in the image plane (in the FOS), assumed to come from some 2D BB face hypothesis $\left(u_{n,o}^{i},v_{n,o}^{i}\right)$, around the mouth.

It is then interesting to obtain further information about that face hypothesis that may give robustness to the validation process commented. This information is going to be the face hypothesis 2D BB size ($S_{n,o}^{i}$). To determine it, the face height of a person $h_{r}^{3D}$ is assumed to be constant and known in the 3D WCS. Thus, it is projected to the FOS through the distance $d_{n,o}^{i}$, as shown in Equation (14):

(14) $$S_{n,o}^{i}=\frac{h_{r}^{3D}\cdot f_{c}}{d_{n,o}^{i}},$$

where $f_{c}$ is the camera focal length.

Once the speakers position hypothesis $\left\{p_{n,o}^{i}\right\}$ are projected from the WCS to the FOS, each particle represents the speaker mouth 2D location as $\left\{{\tilde{p}}_{n,o}^{i}\right\}=\left\{{\left(u_{n,o}^{i},v_{n,o}^{i},S_{n,o}^{i}\right)}^{T}\right\}$.

**Figure 3:**
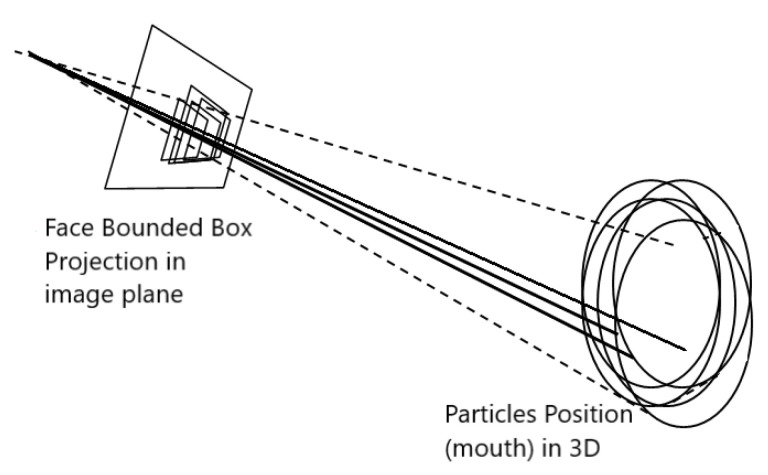
Projections of each mouth hypothesis $\left\{p_{n,o}^{i}\right\}$ in the 3D WCS to its corresponding 2D BB in the FOS $\left\{{\tilde{p}}_{n,o}^{i}\right\}$.

Figure 3 shows a schematic view of the projection mechanism here described.

### 5.2. Appearance-Based Multi-Pose Video Observation Model

For the sake of clarity, the core of the video observation model based on the VJ likelihood, and its characteristics will be explained first. Then the rest of the processes will be detailed.

#### 5.2.1. Viola and Jones Likelihood Model

The VJ likelihood evaluation is made using the probabilistic VJ model described in [44]. This model consists of modifying the standard VJ face detector to obtain face likelihood values. Given an image position ${\tilde{p}}_{n,o}^{i}$, the model applies a cascade of face-trained classifiers returning a likelihood value Ω as in Equation (15):

(15) $$\Omega =\frac{\kappa }{M}\frac{\sum_{m=1}^{\kappa }\left(H_{m}-\theta_{m}\right)}{\sum_{m=\kappa }^{M}m}$$

where κ is the number of stages that the image patch passes through the cascade of classifiers, *M* is the total number of stages, $H_{m}$ is the weight output by the stage *m*, and $\theta_{m}$ is its threshold.

**Figure 4:**
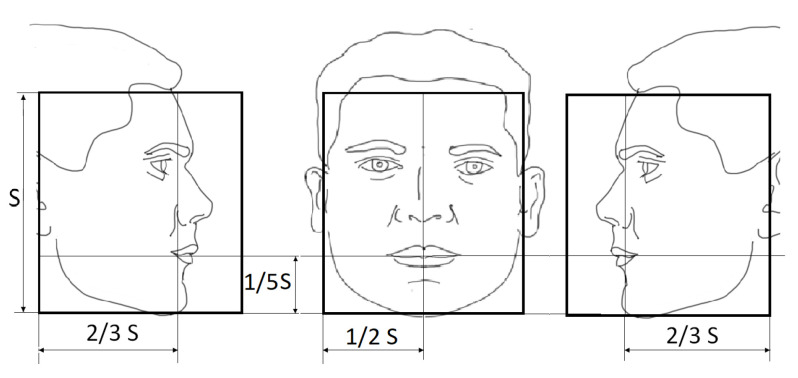
Face templates for the right profile, frontal, and left profile poses.

The likelihood model is applied with three different templates to evaluate different possible face poses in yaw rotations. One template handles frontal faces, and the other two handle left and right profile faces, respectively. Therefore, within the 2D BB, the mouth position where the proposed visual likelihood model is applied is different for each template, as shown in Figure 4.

The approach is flexible enough to allow for other poses to be considered, by, for example, extending the previous templates with in-plane rotations (roll). In this case, the image can be rotated with a given angle (α) in the opposite direction to allow reusing the already trained poses.

In this work, the image and the particles are rotated in both directions (clockwise and counterclockwise). For each direction, the models for the three face pose classifiers (front, right, and left profiles) are applied.

Thus, for each position ${\tilde{p}}_{n,o}^{i}$ in the image, nine response values are obtained, as shown in Equation (16):

(16) $$\Omega \left({\tilde{p}}_{n,o}^{i}\right)=\left[\begin{array}{c} \Omega^{F_{0}}\left({\tilde{p}}_{n,o}^{i}\right)\\ \Omega^{R_{0}}\left({\tilde{p}}_{n,o}^{i}\right)\\ \Omega^{L_{0}}\left({\tilde{p}}_{n,o}^{i}\right)\\ \Omega^{F_{-\alpha }}\left({\tilde{p}}_{n,o}^{i}\right)\\ \Omega^{R_{-\alpha }}\left({\tilde{p}}_{n,o}^{i}\right)\\ \Omega^{L_{-\alpha }}\left({\tilde{p}}_{n,o}^{i}\right)\\ \Omega^{F_{+\alpha }}\left({\tilde{p}}_{n,o}^{i}\right)\\ \Omega^{R_{+\alpha }}\left({\tilde{p}}_{n,o}^{i}\right)\\ \Omega^{L_{+\alpha }}\left({\tilde{p}}_{n,o}^{i}\right) \end{array}\right],$$

where *F*, *R*, and *L* refer to frontal, right, and left profiles, respectively, and their subindexes refer to the angle rotation.

**Figure 5:**
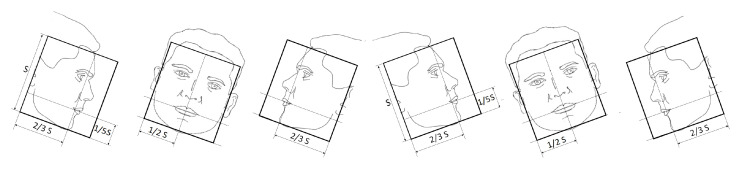
Face templates with in-plane rotations with $\alpha =15^{\circ }$ (counterclockwise, the three on the left, and clockwise the three on the right).

Figure 5 shows the templates for in-plane face rotations: three on the left with clockwise rotation, and three on the right with counterclockwise ones, using $\alpha =15^{\circ }$.

**Figure 6:**
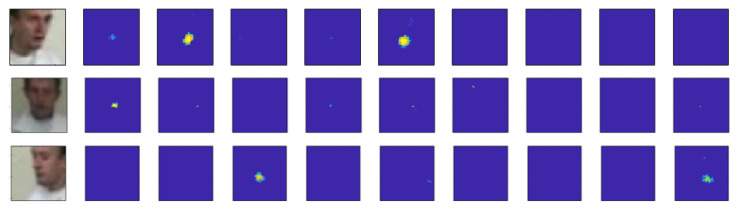
Face likelihood model response for different poses: right at the top row, frontal at the middle row, and left at the bottom row. From left to right in each row: $\Omega^{F_{0}}\left({\tilde{p}}_{n,o}^{i}\right)\;\Omega^{R_{0}}\left({\tilde{p}}_{n,o}^{i}\right)\;\Omega^{L_{0}}\left({\tilde{p}}_{n,o}^{i}\right)\;\Omega^{F_{-\alpha }}\left({\tilde{p}}_{n,o}^{i}\right)\;\Omega^{R_{-\alpha }}\left({\tilde{p}}_{n,o}^{i}\right)\;\Omega^{L_{-\alpha }}\left({\tilde{p}}_{n,o}^{i}\right)\;\Omega^{F_{+\alpha }}\left({\tilde{p}}_{n,o}^{i}\right)\;\Omega^{R_{+\alpha }}\left({\tilde{p}}_{n,o}^{i}\right)\;\Omega^{L_{+\alpha }}\left({\tilde{p}}_{n,o}^{i}\right)$.

The characteristics of the model response are explored in the FOS. Figure 6 shows the responses of the nine templates of the VJ likelihood model for three different face poses. The different templates better respond to faces with poses close to those with similar profiles and rotations. The likelihood responses have higher values around the mouth position for each pose, and more than one template can generate a positive response to the face when the pose is similar.

**Figure 7:**
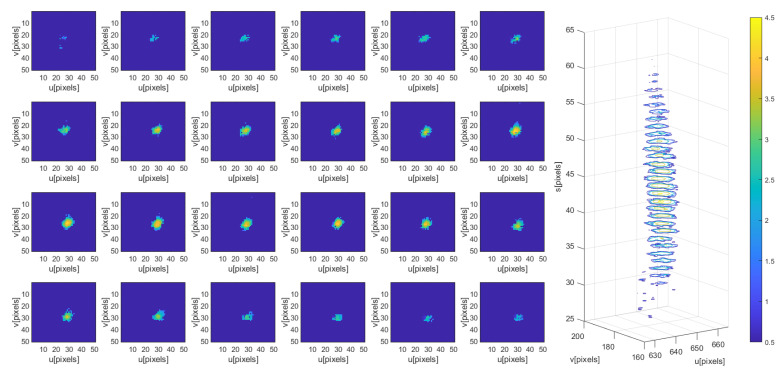
Face likelihoods response at different 2D BB sizes ($S_{n,o}^{i}$) in pixels. (**Left**): likelihood maps. (**Right**): 3D plot of the likelihoods in the FOS $\left(u_{n,o}^{i},v_{n,o}^{i},S_{n,o}^{i}\right)$.

Figure 7 shows that the model response to a face is shaped like a blob in the FOS. This blob-shape-like behavior resembles that of Gaussian behavior. The width of the significant response levels in the (u,v) plane is small, a few pixels in diameter. The width in response to the template size *S* is much larger, several tens of pixels.

#### 5.2.2. Exploration Mechanism of the FOS

One exploration alternative could be to evaluate the likelihood of the particle’s projection on the FOS. However, the peak’s width of the response on the image plane (u,v) is very narrow. This characteristic is right from the point of view of location accuracy, but it increases the possibility that no particle will hit the same region where a peak appears in the FOS. On the other hand, exploring the FOS domain in the whole area occupied by the particles may imply a high computational cost.

To ensure that the region with a peak response is found while keeping computational complexity low, we plan to take advantage of the model’s face response’s redundancy in dimension *S*. Our proposal consists of exploring the volume occupied by particles in the FOS using three slices in the *S* dimension. With the response in these slices, we can estimate a Gaussian shape of the likelihood in the FOS, to finally, weight all particles with the estimated Gaussian.

**Figure 8:**
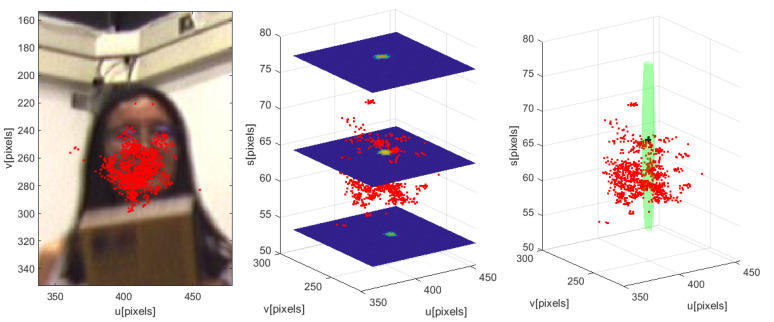
VJ Likelihood Model: Predicted particles (red dots) projected on the image (**left**), the three slices in the *S* dimension (**middle**) with the particles (red dots), and the estimated Gaussian (green blob) along with particles (red dots) in the FOS (**right**).

The three slices or scanning planes are defined, one at the average face size, one γ times larger, and one γ times smaller than the average size: $\left[\bar{S}/\gamma ,\bar{S},\bar{S}\cdot \gamma \right]$. The average face size of particles is computed as ${\bar{S}}_{o}=\frac{1}{N_{P}}\sum_{i=1}^{N_{P}}S_{o}^{i}$. In (u,v), the exploration is restricted to the rectangular area defined by the maximum and minimum particle position values in each dimension $\left[u_{min}:u_{max},v_{min}:v_{max}\right]$. Figure 8 shows the process to weight the particles.

#### 5.2.3. Gaussian Approximation

For each pose, the VJ likelihood is evaluated in the three slices. Next, a threshold $\theta_{VJ}$ is applied to remove points outside the Gaussian pursuit, and the mean $\mu_{n,o}^{\tilde{p}}=\left(\mu_{u},\mu_{v},\mu_{s}\right)$ and covariance matrix $\Sigma_{n,o}^{\tilde{p}}$ of the remaining points are computed.

Next, instead of adding all pose outputs, like in [2,23], we select the best pose ($pose_{best}$) as that with the highest response value (described in Equation (17)), proven to reduce the number of false positives in preliminary experiments.

(17) $$pose_{n,o}^{best}=\underset{pose}{arg max}\left\{\Omega^{pose}\left({\tilde{p}}_{n,o}^{i}\right)\right\},\;with\;pose\in \left\{F_{0}R_{0}L_{0}F_{-\alpha }R_{-\alpha }L_{-\alpha }F_{+\alpha }R_{+\alpha }L_{+\alpha }\right\}$$

Using the centroid’s value $\mu_{n,o}^{\tilde{p}}$, the corresponding pose face BB is projected to the Fg./Bg. image. Finally, poses whose BB have less than 60% of intersection with the foreground area are eliminated, as 60% is the approximated area percentage covered by a face in the templates.

From the remaining poses, that with the greatest number of particles is selected on the set of not eliminated poses, and the observation confidence of the appearance-based model is set to one: $C_{o}^{VJ}=1$. In the case all the poses were eliminated, it will be considered that there is no visual observation with the appearance-based model, setting $C_{o}^{VJ}=0$ and emptying the associated $\mu_{n,o}^{\tilde{p}}$ and $\Sigma_{n,o}^{\tilde{p}}$.

Finally, some adjustment to the covariance matrix is needed. To avoid orientation artifacts in the generated Gaussian model, covariance terms are not considered, setting $\sigma_{uv,o}=\sigma_{us,o}=\sigma_{vs,o}=0$. Also, the horizontal and vertical dispersion values are equalized, taking the minimum of both values $\sigma_{uu,o}=\sigma_{vv,o}=min\left(\sigma_{uu,o},\sigma_{vv,o}\right)$. To prevent a zero variance in the *S* dimension, caused by the case where only one slice had points over the threshold, its minimum value is set to $\sigma_{ss,o}^{min}$, so that $\sigma_{ss,o}\geq \sigma_{ss}^{min}$.

The right image in Figure 8, shows the particles (red), their mean value (black cross), and the standard deviation of the estimated Gaussian as a 3D surface (green).

#### 5.2.4. Occlusion Detection with the VJ Model

After estimating which of the measurements are associated with each target, it is necessary to make sure that multiple targets are not related to the same measurement.

The following procedure is used to avoid this situation:

- For each pair of targets $\left(o,o{}^{\prime }\right)$, the overlap between the exploration spaces (FoVs) of both targets is calculated $\rho =({\left[u_{min}:u_{max},v_{min}:v_{max}\right]}_{o}\cap {\left[u_{min}:u_{max},v_{min}:v_{max}\right]}_{o{}^{\prime }})$.
- If there is overlap (ρ≠∅), the global possible measurements’ dispersion $\sigma_{uu,o}$ for each target (o) in the image plane (u,v) is calculated.
- If any of the targets have high dispersion, it may imply that some of the assigned measurements are from other targets. The threshold value to consider that a target has a high measurement dispersion is calculated as a fraction of the average face size of the particle set ${\bar{S}}_{o}$.
- If the dispersion of any target exceeds this threshold and is not occluded, the Gaussian related to that target observation is recalculated after removing the assigned measurements within the ρ overlapping region. Then, the Euclidean distance between the two centroids of the observations reassigned to each target within the image plane is calculated as $d_{uv}=∥\left(\mu_{n,o}^{\tilde{p}},\mu_{n,o{}^{\prime }}^{\tilde{p}}\right)∥$.
- If this $d_{uv}$ distance is less than two times the dispersion in the image plane ($2\cdot \sigma_{uu,o}$) the decision of which measurement is associated with which target is based on another definition of the distance $d_{uv}$ is defined, using the representation of the predicted particles in the image plane as $d_{uv}=∥\left(\mu_{n,o}^{\tilde{p}},{\bar{p}}_{n,o}\right)∥$. Then again, the observations will be assigned to the target (o) from which the shortest distance $d_{uv}$ is found, and the other target $\left(o^{\prime }\right)$ is declared as occluded, and the related particles are at a shorter distance than the threshold.

The pseudocode of this process is presented in Algorithm 1.

**Figure 9:**
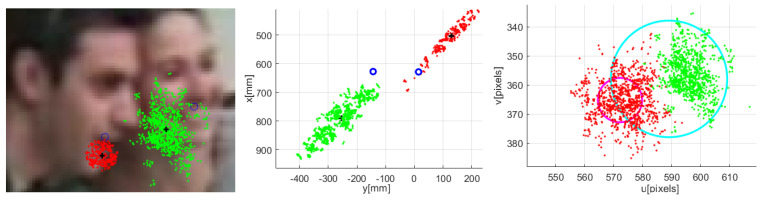
Occlusion situation. Image with particles’ projection (**left**). Top view of 3D position-related particles (**middle**). The same with standard deviation circles (**right**). For target one the particles are in red color, and the standard deviation circle in magenta. For target two the particles are in green color, and the standard deviation circle in cyan.

Figure 9 shows an occlusion situation. In the left graph, the image and the related locations (in the image plane) from the particles representing two targets are shown in red and green colors. The center graph shows a top view of the particle positions in the WCS.

Finally, the right-hand graph shows the same particles’ related location in 2D together with the standard dispersion $\sigma_{uu,o}$, through its representing circle (in magenta and cyan). There, it can be observed that part of the particles representing target number two (green) are being assigned in the observation space to target one (red). It can be thus noticed the particles dispersion, and therefore the occlusion situation.
**Algorithm 1** Occlusions detection in VJ likelihood block.**if** every target pair $o,o{}^{\prime }$ in FoV **then** $\rho \leftarrow ({\left[u_{min}:u_{max},v_{min}:v_{max}\right]}_{o}\cap {\left[u_{min}:u_{max},v_{min}:v_{max}\right]}_{o{}^{\prime }})$         % intersection factor **if** ρ≠∅ **then**  find intersection region, $\left({\left[u_{min},u_{max},v_{min},v_{max}\right]}_{inter}\right)$   **for** $o^{*}\in \left\{o,o^{\prime }\right\}$ **do**    **if** $\sigma_{uu,o*}>{\bar{S}}_{o*}$ and faceIsOccluded(o*)==0 **then**     ${\left[u_{min}:u_{max},v_{min}:v_{max}\right]}_{o*}\leftarrow {\left[u_{min}:u_{max},v_{min}:v_{max}\right]}_{o*}-\rho$     **end if**  **end for**   **if** $d_{uv}=∥\left(\mu_{n,o}^{\tilde{p}},\mu_{n,o{}^{\prime }}^{\tilde{p}}\right)∥<\sigma_{uu,o}$ **then**    **if** $d_{uv}=∥\left(\mu_{n,o{}^{\prime }}^{\tilde{p}},{\bar{p}}_{n,o}\right)∥<d_{uv}=∥\left(\mu_{n,o}^{\tilde{p}},{\bar{p}}_{n,o}\right)∥$ **then**     faceIsOccluded(o)←1 % The target with prediction farthest from the measure is occluded     **for** each particle **do**      **if** $d_{uv}=∥\left(\mu_{n,o{}^{\prime }}^{\tilde{p}},{\tilde{p}}_{n,o}^{i}\right)∥<2\sigma_{uu,o{}^{\prime }}$ **then**       occIdx(i)←1                     % particles are set as occluded      **end if**     **end for**    **else**     $faceIsOccluded(o{}^{\prime })\leftarrow 1$     **for** each particle **do**      **if** $d_{uv}\left(\mu_{n,o}^{\tilde{p}},{\tilde{p}}_{n,o^{\prime }}^{i}\right))<2\sigma_{uu,o}$ **then**       occIdx(i)←1      **end if**     **end for**    **end if**  **end if** **end if****end if**

#### 5.2.5. VJ Likelihood Assignment

The likelihood values assigned to the particles are associated with the Gaussian with mean $\mu_{n,o}^{\tilde{p}}$, covariance matrix $\Sigma_{n,o}^{\tilde{p}}$ and amplitude equal to the maximum observed there $\Omega^{pose_{best}}$ (with $pose_{n,o}^{best}$ as defined in Equation (17)). Likelihood values below the threshold $\theta_{VJ}$ are set to zero. Equation (18) describes such likelihood.

(18) $$l^{VJ}\left({\tilde{p}}_{n,o}^{i}\right)=\left\{\begin{array}{cc} \Omega^{pose_{best}}N\left({\tilde{p}}_{n,o}^{i}|\mu_{n,o}^{\tilde{p}},\;\Sigma_{n,o}^{\tilde{p}}\right)\; & \mathrm{if}\;\Omega^{pose_{best}}N\left({\tilde{p}}_{n,o}^{i}|\mu_{n,o}^{\tilde{p}},\;\Sigma_{n,o}^{\tilde{p}}\right)\geq \theta_{VJ}\\ 0 & \mathrm{otherwise} \end{array}\right.$$

### 5.3. Head Color-Based Likelihood Block

As explained above, in the FOS coordinates, each particle is assigned a BB in the image, corresponding to the face in a frontal pose. To tackle face occlusions, the spatiogram of the 2D BB mouth estimation $BB_{n,o}^{i}$ is calculated as in [17], and replicated in Equation (19). The likelihood will be proportional to the Bhattacharyya coefficient between the histogram associated with each particle i and the spatiogram of the reference face patch image $BB_{n,o}^{fa}$:

(19) $$S\left(BB_{n,o}^{i},BB_{n,o}^{fa}\right)=\sum_{b=1}^{B}\sqrt{r_{i}^{b}r_{fa}^{b}}\left[8\pi \right|\Sigma_{i}^{b}\Sigma_{fa}^{b}{|}^{\frac{1}{4}}N\left(\mu_{i}^{b}|\mu_{fa}^{b},2\left(\Sigma_{i}^{b}+\Sigma_{fa}^{b}\right)\right)],$$

were $r_{i}^{b},\mu_{i}^{b},\Sigma_{i}^{b}$ and $r_{fa}^{b},\mu_{fa}^{b},\Sigma_{fa}^{b}$ are the histogram count, the spatial mean and covariance matrices in the color bin *b* of particle i and in the reference face patch image fa, respectively.

The face is considered to be located by the color model (setting $C_{o}^{col}=1$) if the maximum similarity value exceeds a threshold $\theta_{col}$. In this case, the 75th percentile of the lower likelihoods is discarded, to reduce the dispersion of particles due to the higher dispersion of the color model. Otherwise, we assume that the face is not detected by this color-based strategy (setting $C_{o}^{col}=0$).

Equation (20) describes the final color likelihood proposed $l^{Col}\left({\tilde{p}}_{n,o}^{i}\right)$.

(20) $$l^{Col}\left({\tilde{p}}_{n,o}^{i}\right)=\left\{\begin{array}{cc} S(BB_{n,o}^{i},BB_{n,o}^{fa}) & \mathrm{if}\;max\left(S\left(BB_{n,o}^{i},BB_{n,o}^{fa}\right)\right)\geq \theta_{col}\\ 0 & \mathrm{otherwise} \end{array}\right.$$

The color reference histogram is initialized in the face BB of the first ground-truth frame, considering a frontal pose. This reference model is updated in every iteration when the VJ likelihood delivers a confident value. In this case, the face BB corresponding to the best-detected pose is used.

Like in the VJ likelihood block, with the color model, particles from an occluded target *o*, very close to another target $o^{\prime }$, can be captured by the significant likelihood region of $o^{\prime }$. To avoid this situation, the *o* particles that are very close to $o^{\prime }$ are labeled as occluded. The limit value of closeness is set equal to the diagonal of the average face size of the $o^{\prime }$ target $\sqrt{2}{\bar{S}}_{o^{\prime }}$. This procedure is carried out before evaluating the color-based likelihood.

### 5.4. Correction on Occlusions

The occlusion correction stage is aimed at avoiding hypotheses of one person’s position (target) obtaining a high likelihood if mistaken for another target. Simultaneously, this correction mechanism should not penalize hypotheses in an occluded region, thus allowing the mechanism to keep track of persons passing behind one another in the monitored environment.

The correction globally works as described in Algorithm 2. If a target is occluded and all its related position hypotheses are in an occluded area, all their likelihood values $l^{v}\left({\tilde{p}}_{n,o}^{i}\right)$ are set to $1/N_{P}$. Otherwise, if the target has some position hypotheses in an occluded area and some others in a non-occluded one, the likelihood of those located in the occluded area (occIdx(i)==1) is set to the average of those in a non-occluded one (occIdx(i)≠1).
**Algorithm 2** Occlusions correction pseudocode.**for** every target o **do** **if** target o is occluded **then**  **if** all position hypotheses ${\tilde{p}}_{n,o}^{i}$ are occluded **then**    $l^{v}\left({\tilde{p}}_{n,o}^{i}\right)=1/N_{P}\forall i$   **else**    $l^{v}\left({\tilde{p}}_{n,o}^{i}/occIdx\left(i\right)==1\right)=mean\left(l^{v}\left({\tilde{p}}_{n,o}^{i}/occIdx\left(i\right)\neq 1\right)\right)\forall i$   **end if** **end if****end for**

### 5.5. Foreground vs. Background Segmentation (Fg. vs. Bg.)

The foreground vs. background segmentation procedure starts by subtracting a reference frame (with the environment background, without people) from the given one, in grayscale. After the subtraction, a threshold $\theta_{fg}$ is applied to the resulting difference image, obtaining a binary image $I_{\theta ,fg}$.

Hypotheses in the foreground or outside the camera’s FoV receive a uniformly distributed likelihood value ($U({\tilde{p}}_{n,o}^{i})$). In contrast, those within the FoV but in the background, receive a zero weight, as stated in Equation (21):

(21) $$l^{fg}\left({\tilde{p}}_{n,o}^{i}\right)=\left\{\begin{array}{cc} ∼U({\tilde{p}}_{n,o}^{i}) & \mathrm{if}\;I_{\theta ,fg}\left({\tilde{p}}_{n,o}^{i}\right)==1\vee \notin \mathrm{FoV}\\ ∼0 & \mathrm{otherwise} \end{array}\right\}$$

## 6. Audio Observation Model

In this work, the audio observation model is based on a probabilistic version of SRP-PHAT, proposed in [55]. This model exploits a probabilistic interpretation of the Generalized Cross-Correlation with PHAT transform (GCC-PHAT) between the signals of each pair of microphones. With probabilistic GCC-PHAT, it is possible to associate only one correlation peak to each target. For each time step, the procedure works as follows:

1. The GCC-PHAT for every pair of microphones, and its associated Gaussian model are obtained.
2. SRP-PHAT is computed for every particle position.
3. A Gaussian selection procedure chooses which Gaussian is associated with each target.
4. Finally, the probabilistic SRP-PHAT value is associated with the likelihood of the targets.

**Figure 10:**
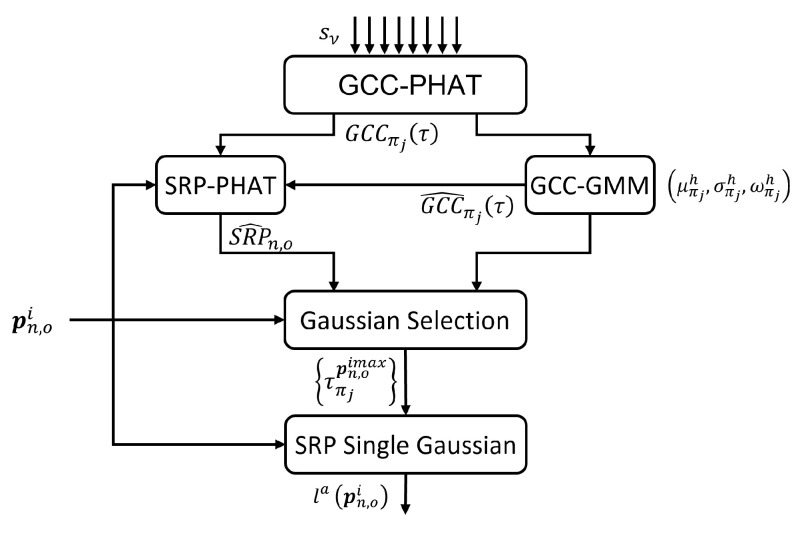
General scheme of the audio likelihood model.

Figure 10 shows the general scheme of the audio observation model.

### 6.1. GCC-PHAT and Gaussian Model

The GCC-PHAT (to ease the notation, we will skip the explicit mention to PHAT when referring to the $GCC{}^{PHAT}$ and $SRP{}^{PHAT}$ functions in the equations) is computed for the signals arriving at each microphone pair $\pi {}_{j}$ (composed of microphones $m_{j_{1}}$ and $m_{j_{2}}$) around the evaluated video frame, as presented in Equation (22):

(22) $$GCC{}_{\pi {}_{j}}\left(\tau \right)=\sum_{k=0}^{N_{f}-1}\Psi_{j_{1}j_{2}}\left[k\right]S_{j_{1}}\left[k\right]S_{j_{2}}^{*}\left[k\right]e^{j\frac{2\pi kf_{s}}{N_{f}}\tau }$$

where $N_{f}$ is the number of discrete frequencies used in the Fourier analysis of the discretized signals captured by the $j_{1}$ and $j_{2}$ microphones (sampled versions of the $s_{j_{1}}\left(t\right)$ and $s_{j_{2}}\left(t\right)$ signals); $S_{j_{1}}\left[k\right]$ and $S_{j_{2}}\left[k\right]$ are the frequency spectra of these signals; and $\Psi_{j_{1}j_{2}}\left[k\right]=\frac{1}{|S_{j_{1}}\left[k\right]S_{j_{2}}^{*}\left[k\right]|}$ is the PHAT filter. τ is the lag variable of the correlation function, associated with the time difference of arrival of the audio signal to the pairs of microphones.

The second step is to model the GCC-PHAT of each microphone pair $\pi {}_{j}$ as a Gaussian Mixture Model (GMM). The main assumption here is that each GCC-PHAT peak is caused by a different acoustic source at a given position, generated by the direct propagation path, by a reverberant echo, or by other noise sources. With this consideration, each peak in the GCC-PHAT function is associated with a Gaussian function in the GMM model described by Equation (23):

(23) $${\hat{GCC}}_{\pi {}_{j}}\left(\tau \right)\approx \sum_{h=0}^{N_{J}-1}\omega {}_{\pi {}_{j}}^{h}N\left(\tau |\mu_{\pi {}_{j}}^{h},\sigma_{\pi {}_{j}}^{h}\right),$$

where $N_{J}$ is the number of peaks detected in the GCC-PHAT function, and $\mu_{\pi {}_{j}}^{h}$, $\sigma_{\pi {}_{j}}^{h}$ and $\omega_{\pi {}_{j}}^{h}$ represent the mean, standard deviation and weights of the $h^{th}$ component of the mixture.

The correlation values are first normalized (making their sum equal to one) and their negative values are set to zero. The GMM parameters ${\left\{\mu_{\pi {}_{j}}^{h},\sigma_{\pi {}_{j}}^{h},\omega_{\pi {}_{j}}^{h}\right\}}_{h=1}^{N_{J}}$ are estimated according to the procedure described in [55].

### 6.2. SRP-PHAT and Gaussian Selection

Once the GMM model is available, the traditional SRP-PHAT formulation can be applied to the position of each particle. Then, the SRP-PHAT value for a given target *o* is calculated as the average $\hat{SRP}$ over all the particles associated with *o*, ${\overset{-}{SRP}}_{n,o}$.

Gaussian selection is applied sequentially to every target, starting from the one with the highest ${\overset{-}{SRP}}_{n,o}$ value. For each target o and microphone pair $\pi {}_{j}$, the set of maximum SRP-PHAT delays $\left\{\tau_{\pi {}_{j}}^{p_{n,o}^{imax}}\right\}$ is evaluated in each Gaussian *h*, and that with the highest value is selected, as shown in Equation (24).

(24) $$p_{n,o}^{imax}=\underset{p_{n,o}^{i}}{arg max}\left(\hat{SRP}\left(p_{n,o}^{i}\right)\right)$$

After the selection, the selected Gaussian is subtracted from the mixture, and the Gaussian selection process continues with all the other targets, in ${\overset{-}{SRP}}_{n,o}$ value decreasing order.

**Figure 11:**
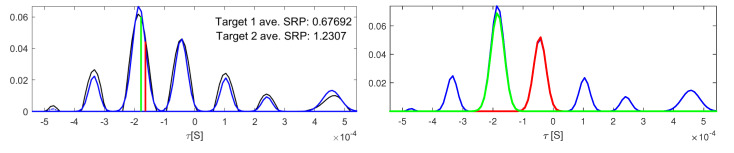
Gaussian selection. In the left graphic: $GCC{}_{\pi {}_{16}}\left(\tau \right)$ in black, and ${\hat{GCC}}_{\pi {}_{16}}\left(\tau \right)$ in blue. In the right graphic: selected Gaussians for target 1 (red) and target 2 (green). The GMM model was generated with $N_{J}=7$.

Figure 11 represents the Gaussian selection process in a frame extracted from sequence seq18-2p-0101 in the AV16.3 dataset, where there were two close speakers. The graphic on the left shows in black the $GCC{}_{\pi {}_{16}}\left(\tau \right)$ function (for the $\pi {}_{16}$ microphone pair) and the calculated Gaussian mixture ${\hat{GCC}}_{\pi {}_{16}}\left(\tau \right)$ in blue, along with the projections of the particles with maximum $\hat{SRP}\left(p_{n,o}^{i}\right)$ for both targets. The graphic on the right highlights the selected Gaussians for each target. In both graphics, target one selected Gaussian appears in red, and target two appears in green.

From Figure 11 it can be observed that both targets obtain close *TDoA* projection for their particles with maximum $\hat{SRP}\left(p_{n,o}^{i}\right)$ value, sharing the same Gaussian. In this case, target 2 obtained the highest value, so it was assigned the associated Gaussian.

### 6.3. Single Gaussian SRP-PHAT Model

The final step to generate a likelihood value from the acoustic information consists of simplifying the SRP-PHAT GMM model, by considering just one Gaussian for each pair of microphones, that with the highest weight value, as shown in Equation (25).

(25) $$\hat{SRP}\left(p_{n,o}^{i}\right)\approx \sum_{j=1}^{N_{Q}}\omega_{\pi {}_{j}}^{*}N\left(\tau |\mu_{\pi {}_{j}}^{*},\sigma_{\pi {}_{j}}^{*}\right)$$

where $\omega_{\pi {}_{j}}^{*}$, $\mu_{\pi {}_{j}}^{*}$ and $\sigma_{\pi {}_{j}}^{*}$ are the parameters associated with the Gaussian component with the highest weight value in Equation (23).

Because of reverberation and low SNR conditions, some speech segments may exhibit low SRP-PHAT values, degrading the quality of the acoustic power maps. To avoid this degradation, we consider the maximum SRP-PHAT value as an indicator of confidence, so that a threshold $\theta_{a}$ is applied to limit the influence of such segments. Finally, the likelihood from the audio information is calculated as described in Equation (26):

(26) $$p\left(z{}_{n,o}^{a}|x_{n,o}^{i}\right)\propto l^{a}\left(p_{n,o}^{i}\right)=\left\{\begin{array}{cc} ∼\hat{SRP}\left(p_{n,o}^{i}\right) & if\max\left(\hat{SRP}\left(p_{n,o}^{i}\right)\right)\geq \theta_{a}\\ ∼U(p_{n,o}^{i}) & otherwise \end{array}\right.$$

## 7. Experimental Setup

The tracking system has been evaluated in three modalities. The first one uses only audio information, the second one uses only video information, and the final one combines the two sources of information in an audiovisual modality.

### 7.1. Datasets

The databases used for system evaluation are the well-known AV16.3 [45], and CAV3D [17] in the state of the art of interest. Both databases are fully labeled, providing the mouth ground-truth location. Synchronization information between the audio and video streams is also available.

#### 7.1.1. AV16.3

AV16.3 was recorded in the *Smart Meeting Room* of the IDIAP research institute [61], in Switzerland, which consists of a 8.2 m × 3.6 m × 2.4 m rectangular room, containing a centrally located 4.8 m × 1.2 m rectangular table, on top of which there are located two circular microphone arrays of radius 0.1 m, each of them composed of eight microphones. The centers of the two arrays are separated 0.8 m, and their coordinates origin are in the middle point between the two arrays. The room is also equipped with three video cameras providing different-angle views of the room. The database contains audio and video data taken by the three video cameras and the two circular microphone arrays. The cameras have a frame rate of 25 f.p.s. (40 ms period) while the audio has been recorded at 16 kHz. The dataset contains sequences grouped in two contexts, a Single Objective Tracking (SOT) one, and a Multiple Objective Tracking (MOT) one.

#### 7.1.2. CAV3D

CAV3D was recorded in the *Bruno Kessler Center in Information and Communication Technology* (FBK-ICT) in Italy, consisting of a 4.77 m × 5.95 m × 4.5 m rectangular room. Sensing was conducted with a monocular color camera co-located with an 8-element circular microphone array placed in the room’s center. Video was recorded at 15 f.p.s. (≈66.7 ms period). The audio sampling rate was 96 kHz. The dataset contains 20 sequences grouped in three contexts, a Single Objective Tracking (SOT) one, another with a single active speaker and a second interfering person (not speaking) (SOT2), and a Multiple Objective Tracking (MOT) one.

#### 7.1.3. Sequences Selection

The experiments were carried out using the same sequences in AV16.3 and CAV3D evaluated in [17,22], two state-of-the-art proposals used for performance comparison. Table 1 shows the AV16.3 selected sequences: seq08, seq11, seq12 for SOT, and seq18, seq19, seq24, seq25 seq30 for MOT. For each sequence, the three cameras’ views were tested independently and combined with the first microphone array, giving a total of nine evaluation sequences in SOT and fifteen in MOT. Table 2 shows the CAV3D selected sequences, which are all the available ones for the SOT and MOT cases, in which we focused our experimental work.

### 7.2. Evaluation Metrics

The evaluation metrics used are the Track Loss Rate (TLR), and the Mean Absolute Error (MAE), as defined in [17]. The TLR is the percentage of frames with a track loss, where a target is considered to be lost if the error exceeds a given threshold. These metrics will be further specified below.

The MAE for 3D is expressed in *m* as in Equation (27):

(27) $$\epsilon {}_{3D}=\frac{1}{N_{F}N_{S}}\sum_{i=1}^{N_{S}}\sum_{n=1}^{N_{F}}\left|\right|{\hat{p}}_{n,i}-r_{n,i}\left|\right|,$$

where $N_{F}$ is the number of frames evaluated, and ${\hat{p}}_{n,i}$ and $r_{n,i}$ are, respectively, the estimated and real positions of source *i* in frame *n*.

To evaluate the TLR in 3D, a target is considered to be lost if the error with respect to the ground-truth is larger than 300 mm. We also use a fine error metric defined as $\epsilon {}_{3D}^{\prime }$, where only the frames where tracking is successful are considered in Equation (27).

For 2D, the MAE in the image plane is expressed in pixels as in Equation (28):

(28) $$\epsilon {}_{2D}=\frac{1}{N_{F}^{\prime }N_{S}^{\prime }}\sum_{i=1}^{N_{S}^{\prime }}\sum_{n=1}^{N_{F}^{\prime }}\left|\right|{\tilde{\hat{p}}}_{n,i}-{\tilde{r}}_{n,i}\left|\right|,$$

where $N_{S}^{\prime }$ are $N_{F}^{\prime }$ are the number of sources and frames, respectively, in which the source position is inside the camera’s FoV. ${\tilde{\hat{p}}}_{n,i}$ and ${\tilde{r}}_{n,i}$ are, respectively, the projection of ${\hat{p}}_{n,i}$ and $r_{n,i}$ in the image plane.

Moreover, for computing the TRL in 2D it is used a threshold of 1/30 of the image diagonal in pixels. As in the 3D case, a fine error metric $\epsilon {}_{2D}^{\prime }$ is also defined, for this 2D TLR threshold.

When comparing different proposals or experimental conditions, we will also calculate the relative performance improvement in all the evaluated metrics, as follows:

(29) $$\Delta_{Alg1}^{Alg2}=100\;\frac{{\mathrm{Metric}}_{Alg1}-{\mathrm{Metric}}_{Alg2}}{{\mathrm{Metric}}_{Alg1}}\;\;\left[\%\right]$$

where Alg1 and Alg2 refer to the algorithms or conditions we are comparing, and ${\mathrm{Metric}}_{Alg}$ refers to the considered Metric calculated using the corresponding Alg. Given that in all the proposed metrics *the lower the better*, a positive result for $\Delta_{Alg1}^{Alg2}$ implies that Alg2 is better than Alg1.

### 7.3. System Configuration

In AV16.3, the audio signals were resampled up to 96 kHz. Also in this database, each image frame was scaled by 2 to adapt them to the VJ OpenCV [62] templates (20×20 pixels) when they are far away from the camera. Also, a lens distortion correction was applied.

For both AV16.3 and CAV3D data, the audio signal pre-processing starts with a pre-emphasis filter ($H\left(z\right)=1-0.98z^{-1}$) to enhance high-frequency content. After filtering a segment of 8192 samples (≈85.3ms), flattop weighted windows are applied to the signal, with a window shift value equal to the video frame rate (25 f.p.s. in AV16.3 and 15 f.p.s. in CAV3D). Thus, there is one audio segment associated with each video frame. Moreover, the Fourier filter size has been selected equal to the signal window size (8192).

Regarding the algorithm parameters needed in the proposal as described in Section 5 and Section 6, all of them were tuned on a small subset composed of additional sequences from the AV16.3 dataset, except for the Fg./Bg. Segmentation, in which the tuning was carried out using the extra sequence seq21 from CAV3D.

These are the final values in the experimental setup: The face size in 3D was set to a fixed value of $h_{r}^{3D}=17$ cm. The appearance-based likelihood threshold $\theta_{VJ}$, was set to 0.5. The γ face size change factor for slice exploration was set to γ=1.2. The minimum dispersion in the *S* dimension was fixed to $\sigma_{ss}^{min}=10$. The color spatiogram likelihood threshold was set to $\theta_{col}=0.6$. The Fg./Bg. Segmentation gray scale intensity threshold was set to $\theta_{fg}=80$ for AV16.3 and $\theta_{fg}=30$ for CAV3D. The acoustic power threshold $\theta_{a}$ was set to 0.8. The model parameters $\bar{v}=1$ ms${}^{-1}$ and β=10 s${}^{-1}$ used in [60,63] have shown good results, and thus they are the ones we applied in this work. The PF algorithm used was the SIR with $N_{P}=1000$ particles per target.

## 8. Results and Discussion

In this section, different results, both quantitative and qualitative, are included to demonstrate the contributions of the GAVT global proposal and its processes in the audiovisual MOT objective.

As mentioned in Section 2, the AV16.3 and CAV3D datasets are used for the experimental work, due to their application in the most important state-of-the-art related works [17,20].

Therefore two different sections are here included to analyze such results: a first one in which the multimodality of the proposal is evaluated and discussed; and a second one in order to compare them with the best proposals in the state-of-the-art on the selected datasets [20].

In the tables within this section, we will provide values for TLR, $\epsilon {}_{2D}$, $\epsilon {}_{2D}^{\prime }$, $\epsilon {}_{3D}$, $\epsilon {}_{3D}^{\prime }$, segregated for SOT and MOT partitions, and their average value. In all cases, we explicitly include the average of the standard deviation of the metric (±σ), given that we carried out ten runs per sequence due to the probabilistic nature of the GAVT proposal. We will also include information on the modality being used, either audio only (A for short), video only (V for short), or audiovisual (AV for short). Finally, to quickly identify the experimental conditions of each table with results, in the first row we state the dataset used (AV16.3 or CAV3D), and if the metrics are in the image plane (2D) of in the three-dimensional space (3D), also including the modality being used. The comparison between modalities and algorithms will be done using the $\Delta_{alg1}^{alg2}$ metric defined in the previous section. In all cases, the best results across metrics will be highlighted with a green background in the corresponding cell.

### 8.1. Audiovisual Combination Improvements

We first present the contribution of audiovisual tracking versus individual audio-only and video-only modalities on the AV16.3 database sequences. We did not make this comparison in the CAV3D dataset as in that one there are several sequences in which the speakers leave the camera’s FoV for a certain time, so that the video-only modality could not be compared equally with the other two modalities within such dataset.

The results of our GAVT proposal using the audio-only (A), video-only (V) and audiovisual (AV) modalities are shown in Table 3 and Table 4 for the 2D and 3D metrics, respectively, as well as the relative performance improvements comparison from A to AV modalities ($\Delta_{A}^{AV}$), and from V to AV ($\Delta_{V}^{AV}$).

The obtained results clearly show that for both the SOT and MOT tasks, the audiovisual combination outperforms its monomodal counterparts. As expected, the visual modality is far better than the audio modality, but in all cases, the audiovisual combination contributes to improved results.

In the 2D case, the average MAE is strongly improved when combining the audio and video modalities, with similar improvements for the SOT and MOT tasks (86.2% and 87%, respectively), with an average improvement of 86.7%. The improvements of the audiovisual modality as compared with the video-only one are still relevant, being 37.6% on average. When considering the fine MAE, the audiovisual modality does not improve the visual-only modality, with minor degradation of −2.0% on average, which is not significant (especially considering the *non-linear* characteristic of this metric), with errors below 3.35 pixels in all cases.

In the 3D case, the improvements of the audiovisual modality are consistent across all metrics, with MAE of 18 cm on average, and very relevant improvements of up to 50.03% as compared with the visual-only modality.

Also as expected, the results for the SOT task are better than those for the MOT task. For example, in the audiovisual modality, with SOT 2D MAE of 5.12 pixels vs. MOT 2D MAE of 7.4. In the 3D case, the 3D MAE for SOT is 15 cm vs. 21 cm in the MOT task.

For the SOT task, the highest relative improvement in the comparison between audiovisual and audio-only modalities was found for sequence seq11 camera 2, with a 79%, and the lowest relative improvement happened for sequence seq08 camera 1 with a 25%. We will further discuss these two extreme cases.

**Figure 12:**
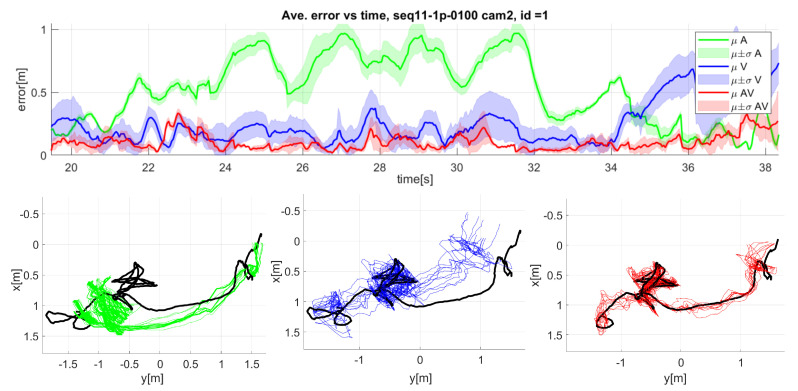
Detailed results for seq11 camera 2: Mean and standard deviation of error over time (**top** graphic), and top view of the speaker trajectory (**bottom** graphics). Green: audio only, blue: video only, red: audiovisual, black: ground truth).

The top part of Figure 12 shows the mean (dark line) and standard deviation (light color area) of the 3D error over time for the seq11 sequence camera 2. The bottom part of Figure 12 shows the top view of the tracked trajectories for each of the modalities.

Analyzing the errors in 2D (Table 3), where the tracked positions are reprojected to the camera image plane, we can see that the video-only modality is very accurate, with an error of 5.78 pixels, and close to the audiovisual solution with 5.12 pixels, while the audio-only modality presents a much higher error of 37.9 pixels.

From comparing 2D and 3D errors, we can observe that the video modality errors come principally from the estimation of depth (see the bottom part of Figure 12), so that we can interpret that the proposed multi-pose face model can accurately locate the mouth in the image plane domain.

From the results of sequence seq11 camera 2 in Figure 12, it can be observed that the audio modality tracker presents significant errors for most of the sequence. This error can be observed in the trajectories (see bottom left graphic in Figure 12) where the system overestimates the distance from the target to the microphone array, especially when the target is far from it. The video-only tracker presents a better estimation in the first part of the sequence but underestimates the distance at the end of the sequence (see bottom middle graphic in Figure 12) when the target is farthest from the camera. Observing the audiovisual trajectories (see bottom right graphic in Figure 12), both modalities compensate for each other errors, presenting an intermediate estimation that leads to improved results.

**Figure 13:**
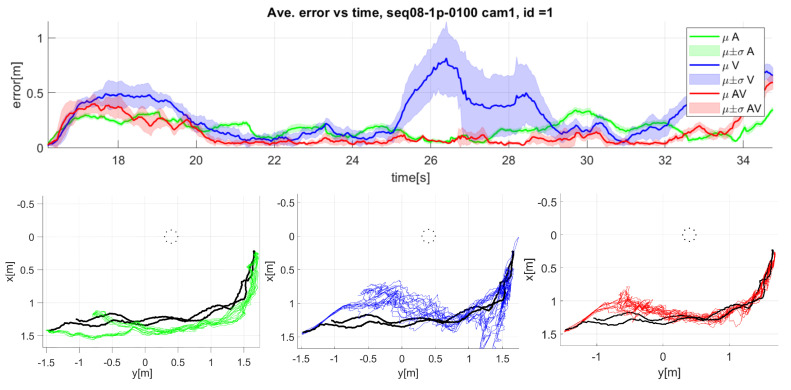
Detailed results for seq08 camera 1: Mean and standard deviation of error over time (**top** graphic), and top view of the speaker trajectory (**bottom** graphics). Green: audio only, blue: video only, red: audiovisual, black: ground truth). The dotted circle represents the microphone array.

Figure 13 shows the detailed results for sequence seq08 camera 1, where it can be observed that the audio-only modality present low errors during the whole sequence (bottom left graphic), while the video-only modality fails on the estimation of depth with important errors (bottom middle graphic). Although the video-only modality presents more relevant errors than the audio-only one, the audiovisual combination improves again the monomodal tracking options.

**Figure 14:**
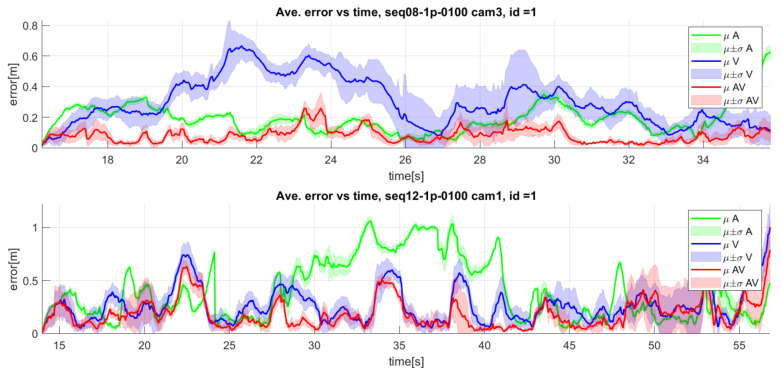
Additional detailed results for sequences seq08 camera 3 and seq12 camera 1: Mean and standard deviation of error over time. Green: audio only, blue: video only, red: audiovisual, black: ground truth).

This behavior of audiovisual integration usually works successfully even when the video performance is lower than the audio. As an example, the upper graphic of Figure 14 shows the 3D MAE variation along time for sequence seq08 camera 3, where the video modality shows higher errors than the audio-only modality, especially in the first half of the sequence. The audiovisual fusion is again able to obtain results that improve the monomodal versions. The lower graphic of Figure 14 shows another case in which the audio modality presents higher errors than the video modality in the central part of the sequence, and the audiovisual fusion compensates for such behavior.

For the MOT task, the audio modality significantly increases its errors, up to 68 cm on average, while the video modality obtained a result of 39 cm. This performance decrease in the audio tracking modality could be explained by targets being interchanged between them.

**Figure 15:**
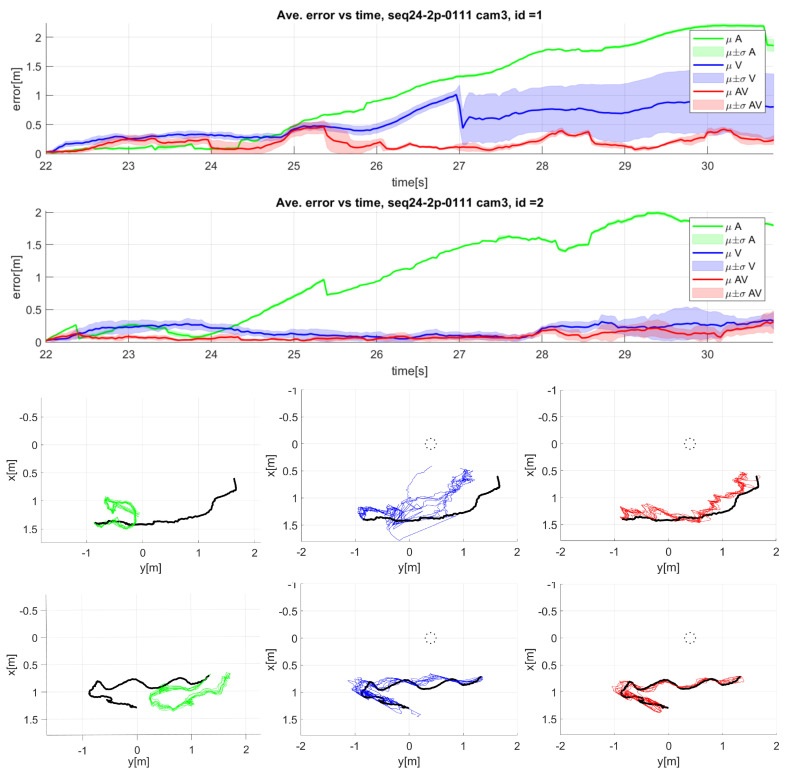
Detailed results for sequence seq24 camera 3: Mean and standard deviation of error over time for the tracking of two speakers (top graphics), and top view of the 3D trajectories of both speakers (bottom graphics). Green: audio only, blue: video only, red: audiovisual, black: ground truth. Speaker one graphics are above those of speaker two. The dotted circle represents the microphone array.

Regarding the detailed analysis for the MOT task, Figure 15 shows an example for sequence seq24 camera 3. The top part of Figure 15 shows the mean (dark line) and standard deviation (light color area) of the 3D error over time for the seq24 sequence camera 3. The bottom part of Figure 15 shows the top view of the tracked trajectories for each of the modalities. In this sequence, there were two speakers, so two sets of graphics are shown.

In this case, the audio-only modality (bottom left graphics of Figure 15) only shows good results in the initial part of the sequence. The video-only modality (bottom middle graphics of Figure 15) exhibits good results for speaker 2, but not so good for speaker one, but the combination of both modalities (bottom right graphics of Figure 15) can compensate the errors, achieving good results even for speaker 1.

**Figure 16:**
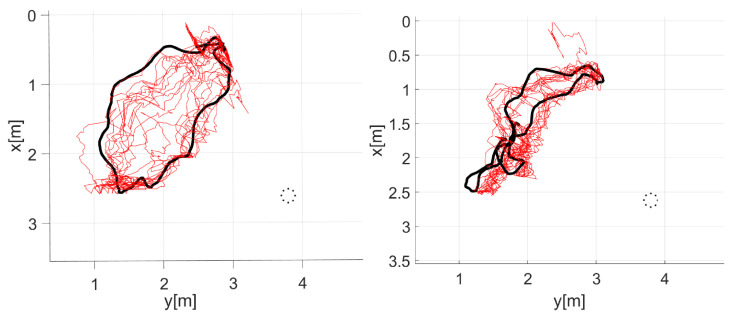
Top view of the 3D trajectories for the two speakers in the seq22 camera 5 (red: audiovisual, black: ground truth). The dotted circle represents the microphone array.

In the MOT task, the average 3D errors are 40% higher compared to the SOT case. We observed that most large errors were associated with instances where one target was lost because of a missing face and the difficulty to recover it, similar to the difficulties encountered in the SOT task. Nevertheless, our occlusion detection and handling mechanisms proved effective in preventing particle interchanges, leading to good results. As an example, in sequence seq22 (see Figure 16), we can see the successful tracking of two targets walking in circular patterns in front of the camera.

**Figure 17:**
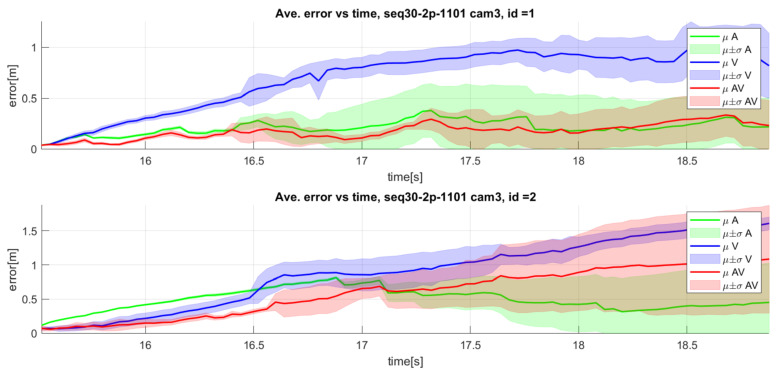
Detailed results for sequence seq30 camera 3: Mean and standard deviation of error over time. **Top**: speaker 1; **Bottom**: speaker 2. Green: audio only, blue: video only, red: audiovisual, black: ground truth).

We can also find examples of audiovisual integration not being able to successfully compensate for the impact of a modality performing badly. Figure 17 illustrates such a scenario, where the system correctly integrates the audio and video modalities for speaker 1 (top graphic), resulting in improved tracking accuracy. However, for speaker 2 (bottom graphic), the integration improves the results as compared with the video-only modality, but grows worse as compared with the audio-only modality toward the end of the sequence.

### 8.2. Comparison with the State-of-the-Art

In this section, we will compare our GAVT proposal with that of [17] (which we will refer to as AV3T) that represents state-of-the-art performance in the AV16.3 and CAV3D datasets within our experimental design.

Table 5 and Table 6 show the 2D and 3D performance metrics of the GAVT and AV3T systems on the AV16.3 dataset, for the audio-only (A) and video-only (V) modalities. The tables also include the relative performance improvements of GAVT as compared with AV3T ($\Delta_{\mathrm{AV}3T}^{\mathrm{GAVT}}$).

From these results, it is clear that our audio-only method performs worse than that proposed by Qian since we did not use height information from the video (−51% performance improvement for the average 2D MAE and −38% for the average 3D MAE).

It’s also clear that our video-only tracker outperforms AV3T in the MAE metrics (23% for both the average 2D and 3D MAEs). This result shows that our proposed pose-dependent visual appearance model works better to localize the mouth than using the generic face detection with a mouth position estimation based on the aspect ratio proposed in [17].

Table 7 and Table 8 show again, for the AV16.3 dataset, the 2D and 3D performance metrics for the audiovisual modality (AV) of the GAVT and the AV3T systems, as well as the improvements of our proposal against AV3T ($\Delta_{\mathrm{AV}3T}^{\mathrm{GAVT}}$).

Our audiovisual method GAVT presents significant improvements as compared with AV3T, for both the 2D and 3D metrics, and for both the SOT and MOT tasks. More important error reductions were found in the 2D MAE metrics (up to almost 70% relative improvement in 2D MAE), while the average relative improvement in 3D MAE is 4.2% at the expense of a performance decrease of −9.4% for the 3D TLR.

The results on the CAV3D dataset for the full system with audio and video integration are presented in Table 9 and Table 10. The performance improvements we achieve are clear for all the 3D metrics and for both the SOT and MOT tasks, reaching an average of 11.5% in the 3D MAE. However, for the 2D metrics, we are only able to improve the average MAE metrics, up to 2.6% in 2D MAE, with improvements in MOT, but not in SOT. It is worth mentioning here that the most relevant metric for our purposes is the 3D MAE, as we are interested in precise 3D localization in the given environment.

It is worth mentioning that our method does not use a face detector and in a context where speakers do not look at the camera for a while, go out of the FoV, or there may be no visible face, particles may move away from the actual target (lose the target) and it is more difficult to recover the location of the speaker. In other words, without a face detector, which scans each frame of the entire image, it is more difficult to recover from a target loss. Despite that, our proposal successfully solves the audiovisual 3D localization task, improving the AV3T system in 3D MAE performance for both the AV16.3 and CAV3D datasets.

### 8.3. Limitations of the GAVT Proposal

We consider that the two databases, AV16.3 and CAV3D, cover a wide range of situations that can be encountered in an intelligent space (different environment sizes, sensor configurations, camera resolutions, speakers moving in and out of the camera’s FoV, etc.). However, there are characteristics of GAVT that may limit its performance in additional situations that may arise in a real-world context.

We can first refer to the presence of different head sizes, which will impact the estimation of the distance from cameras to the speaker: For example, smaller heads (such as children’s) will lead to an overestimation of this distance (given our assumption of an average adult head size).

We can also consider the effect of larger environments, which may first increase the number of possible speakers, thus increasing the difficulty of the task; and second increase the occurrence of situations in which any speaker will not be visible in the camera’s FoV, mainly if the remain silent for long periods of time. In these two situations, the algorithm will have no information to track for a long time, and the particles may drift in the direction from the speaker’s last known position. In these cases, a detection mechanism would be necessary, based on audio and/or video information, to reinsert particles once the speaker starts talking or appears in the camera’s FoV. Larger environments and a higher number of speakers will also require an increase in the particle number, which at its time, may lead to a relevant increase in computational complexity.

## 9. Conclusions and Future Work

In this paper, we have proposed a robust and precise system to track a known number of multiple speakers in a 3D smart space, combining audio and video information. The system uses particle filters with an ad hoc designed audiovisual probabilistic observation model. The visual likelihood model is based on VJ detectors with a pose-dependent strategy that improves the mouth location estimation in 2D and 3D. Additionally, we adopt a specific mechanism to handle MOT tasks and avoid target interference by exploiting the likelihood dispersion effect. The audio likelihood model uses a probabilistic version of SRP, adopting a refined peak selection strategy to avoid target interference, based on the joint distribution of all pairs of microphones. The final fusion model assumes statistical independence of both modalities, so that the audiovisual probability results from the product of audio and video probability density functions.

In the AV16.3 dataset, our audiovisual system proposal shows average relative improvements in 2D mouth localization, of 86.7% and 37.6% over the audio and visual counterparts, respectively. In 3D localization, the improvements were 66.1% and 50.3%. This demonstrates that the proposed audiovisual likelihood combination significantly improves the monomodal counterparts tracking results. When compared to the state-of-the-art, in 2D and 3D metrics, the proposed system presents improved results in the visual and audiovisual modalities for both the SOT and MOT tasks. In the AV16.3 dataset, the 2D average relative improvement is 23% for the visual modality and 69.7% for the audiovisual case, while the 3D improvements are 23% for the visual modality and 4.2% for the audiovisual one. For the audiovisual modality and the CAV3D dataset, the 2D average improvement is 2.6%, while the 3D improvements are 11.5%, rising to 18.1% in the more difficult MOT task. The better results in 2D show that the proposed pose-dependent face model gives a better adapted likelihood of finding the mouth inside the face, in the image plane, as compared with state-of-the-art proposals. The 3D results are consistently better.

The most important errors in the experiments here described are derived from bad depth estimations and target recovery with the visual likelihood model after a target is lost. As a global conclusion, the audiovisual system here proposed and described has been demonstrated to successfully handle occlusions for MOT tasks, and significantly improve state-of-the-art results in a challenging audiovisual tracking context like CAV3D.

In future work, we plan to focus on new alternatives to decrease the depth estimation errors, being the main error source. For this purpose, we plan to improve the head pose information. Rather than a discrete set of possible poses, we will evaluate a continuous estimation of the three head pose angles using recent deep learning-based head pose estimators. The challenge in this case will be the use of head pose estimation algorithms in low-resolution faces, present in the AV16.3 sequences. We will also combine the proposed likelihood model with a face detector, to solve target losses. To deal with long target losses, we will include a birth and death hypotheses mechanism that will also help to handle tracking a variable and an unknown number of speakers.

## Figures and Tables

**Table 1:** Brief descriptions of the sequences in the AV16.3 dataset.

Type	Sequence ID	Description	Time Length (MM:SS)
SOT	seq08	Moving speaker facing the array, walking backward and forward once	00:22.28
SOT	seq11	Moving speaker facing the array, doing random motions	00:30.76
SOT	seq12	Moving speaker facing the array, doing random motions	00:48.16
		**Total SOT**	01:41.20
MOT	seq18	Two moving speakers facing the array, speaking continuously, growing closer to each other, then further apart twice, the first time seated and the second time standing	00:57.00
MOT	seq19	Two standing speakers facing the array, speaking continuously, growing closer to each other, then further from each other	00:22.8
MOT	seq24	Two moving speakers facing the array, speaking continuously, walking backward and forward, each speaker starting from the opposite side and occluding the other one once	00:47.96
MOT	seq25	Two moving speakers greeting each other, discussing, then parting, without occluding each other	00:55.72
MOT	seq30	Two moving speakers, both speaking continuously, walking backward and forward once, one behind the other at a constant distance	00:22.04
		**Total MOT**	03:56.28
		**TOTAL ALL**	05:37.48

**Table 2:** Brief descriptions of the sequences in the CAV3D dataset.

Type	Sequence ID	Description	Time Length (MM:SS)
SOT	seq06	Female moving along a reduced area inside the room.	00:52.10
SOT	seq07	Male moving along a reduced area inside the room	00:58.97
SOT	seq08	Male moving along a reduced area inside the room	01:10.02
SOT	seq09	Male moving along a reduced area inside the room within noisy situations	00:50.43
SOT	seq10	Male moving along a reduced area inside the room within noisy situations	00:50.05
SOT	seq11	Male moving along a reduced area inside the room within clapping/noising and bending/sitting situations	01:10.61
SOT	seq12	Male moving along a reduced area inside the room within clapping/noising and bending/sitting situations	01:28.87
SOT	seq13	Male moving along the whole room	01:24.22
SOT	seq20	Male moving along a reduced area inside the room	00:46.46
		**Total SOT**	9:31.73
MOT	seq22	A female and a male moving along the room simultaneously speaking	00:39.55
MOT	seq23	A female and a male moving along the room simultaneously speaking	01:04.04
MOT	seq24	A female and a male moving along the room simultaneously speaking	01:09.46
MOT	seq25	Two females and a male moving along the room simultaneously speaking	01:02.46
MOT	seq26	Two females and a male moving along the room simultaneously speaking	00:36.48
		**Total MOT**	04:31.99
		**TOTAL ALL**	14:03.72

**Table 3:** Performance scores of GAVT on AV16.3. Average TLR [%] and MAE in 2D [pixels] per modality (A: audio-only, V: video-only, and AV: audiovisual), and summary comparison ($\Delta_{A}^{AV}$: improvement from A to AV, $\Delta_{V}^{AV}$: improvement from V to AV). The best results across metrics are highlighted with a green background.

		GAVT on AV16.3 in 2D (A, V, AV and Comparison)				
		A	V	AV	$\Delta_{A}^{\mathrm{AV}}$	$\Delta_{V}^{\mathrm{AV}}$
SOT	TLR	65.35 ± 3.50	6.42 ± 3.54	5.19 ± 2.55	92.1%	19.2%
	$\epsilon {}_{2D}$	37.09 ± 2.53	5.78 ± 2.11	5.12 ± 1.42	86.2%	11.4%
	$\epsilon {}_{2D}^{\prime }$	8.98 ± 0.41	3.35 ± 0.12	3.35 ± 0.10	62.6%	−0.1%
MOT	TLR	74.56 ± 6.87	20.19 ± 7.49	9.55 ± 7.26	87.2%	52.7%
	$\epsilon {}_{2D}$	57.01 ± 12.12	14.29 ± 7.08	7.40 ± 5.46	87.0%	48.2%
	$\epsilon {}_{2D}^{\prime }$	8.39 ± 0.80	3.15 ± 0.33	3.27 ± 0.32	61.0%	−4.0%
Avg	TLR	69.95 ± 5.18	13.31 ± 5.51	7.37 ± 4.91	89.5%	44.6%
	$\epsilon {}_{2D}$	47.05 ± 7.32	10.03 ± 4.60	6.26 ± 3.44	86.7%	37.6%
	$\epsilon {}_{2D}^{\prime }$	8.69 ± 0.60	3.25 ± 0.22	3.31 ± 0.21	61.9%	−2.0%

**Table 4:** Performance scores of GAVT on AV16.3. Average TLR [%] and MAE in 3D [m] per modality (A: audio-only, V: video-only, and AV: audiovisual), and summary comparison ($\Delta_{A}^{AV}$: improvement from A to AV, $\Delta_{V}^{AV}$: improvement from V to AV). The best results across metrics are highlighted with a green background.

		GAVT on AV16.3 in 3D (A, V, AV and Comparison)				
		A	V	AV	$\Delta_{A}^{\mathrm{AV}}$	$\Delta_{V}^{\mathrm{AV}}$
SOT	TLR	43.80 ± 2.78	46.76 ± 6.14	12.05 ± 3.84	72.5%	74.2%
	$\epsilon {}_{3D}$	0.37 ± 0.02	0.33 ± 0.04	0.15 ± 0.01	59.4%	54.6%
	$\epsilon {}_{3D}^{\prime }$	0.17 ± 0.01	0.15 ± 0.01	0.10 ± 0.01	38.8%	29.3%
MOT	TLR	59.88 ± 9.07	46.15 ± 9.58	19.78 ± 11.0	67.0%	57.1%
	$\epsilon {}_{3D}$	0.68 ± 0.15	0.39 ± 0.09	0.21 ± 0.08	69.7%	46.7%
	$\epsilon {}_{3D}^{\prime }$	0.16 ± 0.02	;0.15 ± 0.02	0.11 ± 0.01	26.1%	24.2%
Avg	TLR	51.84 ± 5.93	46.46 ± 7.86	15.92 ± 7.43	69.3%	65.7%
	$\epsilon {}_{3D}$	0.52 ± 0.09	0.36 ± 0.06	0.18 ± 0.12	66.1%	50.3%
	$\epsilon {}_{3D}^{\prime }$	0.16 ± 0.01	0.15 ± 0.02	0.11 ± 0.01	32.7%	26.7%

**Table 5:** Performance scores of AV3T and GAVT on AV16.3. Average TLR [%] and MAE in 2D [pixels] for the audio-only (A) and video-only (V) modalities, and summary improvements comparison of GAVT to AV3T ($\Delta_{\mathrm{AV}3T}^{\mathrm{GAVT}}$). The best results across metrics are highlighted with a green background.

		GAVT, AV3T and Comparison, on AV16.3 in 2D (A and V)					
		A			V		
		AV3T	GAVT	$\Delta_{\mathrm{AV}3T}^{\mathrm{GAVT}}$	AV3T	GAVT	$\Delta_{\mathrm{AV}3T}^{\mathrm{GAVT}}$
SOT	TLR	48.10 ± 6.00	65.35 ± 3.50	−36%	9.00 ± 1.90	6.42 ± 3.54	29%
	$\epsilon {}_{2D}$	24.10 ± 5.70	37.09 ± 2.53	−54%	8.20 ± 1.10	5.78 ± 2.11	30%
	$\epsilon {}_{2D}^{\prime }$	7.60 ± 0.50	8.98 ± 0.41	−18%	5.30 ± 0.10	3.35 ± 0.12	37%
MOT	TLR	56.60 ± 9.40	74.56 ± 6.87	−32%	15.50 ± 9.00	20.19 ± 7.49	−30%
	$\epsilon {}_{2D}$	38.40 ± 9.20	57.01 ± 12.1	−48%	17.90 ± 8.80	14.29 ± 7.08	20%
	$\epsilon {}_{2D}^{\prime }$	7.70 ± 0.90	8.39 ± 0.80	−9%	5.10 ± 0.40	3.15 ± 0.33	38%
Avg	TLR	52.35 ± 7.70	69.95 ± 5.18	−34%	12.25 ± 5.45	13.31 ± 5.51	−9%
	$\epsilon {}_{2D}$	31.25 ± 7.45	47.05 ± 7.32	−51%	13.05 ± 4.95	10.03 ± 4.60	23%
	$\epsilon {}_{2D}^{\prime }$	7.65 ± 0.70	8.69 ± 0.60	−14%	5.20 ± 0.25	3.25 ± 0.22	38%

**Table 6:** Performance scores of AV3T and GAVT on AV16.3. Average TLR [%] and MAE in 3D [m] for the audio-only (A) and video-only (V) modalities, and summary improvements comparison of GAVT to AV3T ($\Delta_{\mathrm{AV}3T}^{\mathrm{GAVT}}$). The best results across metrics are highlighted with a green background.

		GAVT, AV3T and Comparison, on AV16.3 in 3D (A and V)					
		A			V		
		AV3T	GAVT	$\Delta_{\mathrm{AV}3T}^{\mathrm{GAVT}}$	AV3T	GAVT	$\Delta_{\mathrm{AV}3T}^{\mathrm{GAVT}}$
SOT	TLR	34.90 ± 8.90	43.80 ± 2.78	−26%	52.70 ± 5.50	46.76 ± 6.14	11%
	$\epsilon {}_{3D}$	0.28 ± 0.01	0.37 ± 0.02	−30%	0.41 ± 0.01	0.33 ± 0.04	20%
	$\epsilon {}_{3D}^{\prime }$	0.15 ± 0.01	0.17 ± 0.01	−14%	0.16 ± 0.10	0.15 ± 0.01	8%
MOT	TLR	44.90 ± 1.20	59.88 ± 9.07	−33%	56.30 ± 9.80	46.15 ± 9.58	18%
	$\epsilon {}_{3D}$	0.48 ± 0.12	0.68 ± 0.15	−42%	0.52 ± 0.11	0.39 ± 0.09	26%
	$\epsilon {}_{3D}^{\prime }$	0.15 ± 0.02	0.16 ± 0.02	−4%	0.15 ± 0.02	0.15 ± 0.02	−1%
Avg	TLR	39.90 ± 5.05	51.84 ± 5.93	−30%	54.50 ± 7.65	46.46 ± 7.86	15%
	$\epsilon {}_{3D}$	0.38 ± 0.06	0.52 ± 0.09	−38%	0.47 ± 0.06	0.36 ± 0.06	23%
	$\epsilon {}_{3D}^{\prime }$	0.15 ± 0.02	0.16 ± 0.01	9%	0.16 ± 0.15	0.15 ± 0.02	3%

**Table 7:** Performance scores of AV3T and GAVT on AV16.3. Average TLR [%] and MAE in 2D [pixels] for the AV modality, and summary improvements comparison of GAVT to AV3T ($\Delta_{\mathrm{AV}3T}^{\mathrm{GAVT}}$). The best results across metrics are highlighted with a green background.

		GAVT, AV3T and Comparison (AV), on AV16.3 in 2D		
		AV3T	GAVT	$\Delta_{\mathrm{AV}3T}^{\mathrm{GAVT}}$
SOT	TLR	8.50 ± 3.60	5.19 ± 2.55	38.9%
	$\epsilon {}_{2D}$	16.50 ± 8.60	5.12 ± 1.42	69.0%
	$\epsilon {}_{2D}^{\prime }$	12.20 ± 0.30	3.35 ± 0.10	72.5%
MOT	TLR	11.20 ± 5.90	9.55 ± 7.26	14.7%
	$\epsilon {}_{2D}$	24.80 ± 23.7	7.40 ± 5.46	70.2%
	$\epsilon {}_{2D}^{\prime }$	10.10 ± 0.60	3.27 ± 0.32	67.6%
Avg	TLR	9.85 ± 4.75	7.37 ± 4.91	25.2%
	$\epsilon {}_{2D}$	20.65 ± 16.1	6.26 ± 3.44	69.7%
	$\epsilon {}_{2D}^{\prime }$	11.15 ± 0.45	3.31 ± 0.21	70.3%

**Table 8:** Performance scores of AV3T and GAVT on AV16.3. Average TLR [%] and MAE in 3D [m] for the AV modality, and summary improvements comparison of GAVT to AV3T ($\Delta_{\mathrm{AV}3T}^{\mathrm{GAVT}}$). The best results across metrics are highlighted with a green background.

		GAVT, AV3T and Comparison (AV), on AV16.3 in 3D		
		AV3T	GAVT	$\Delta_{\mathrm{AV}3T}^{\mathrm{GAVT}}$
SOT	TLR	13.30 ± 4.30	12.05 ± 3.84	9.4%
	$\epsilon {}_{3D}$	0.16 ± 0.20	0.15 ± 0.15	7.4%
	$\epsilon {}_{3D}^{\prime }$	0.11 ± 0.10	0.10 ± 0.01	5.1%
MOT	TLR	15.80 ± 8.90	19.78 ± 11.0	−25.2%
	$\epsilon {}_{3D}$	0.21 ± 0.07	0.21 ± 0.08	1.8%
	$\epsilon {}_{3D}^{\prime }$	0.11 ± 0.01	0.11 ± 0.01	−4.4%
Avg	TLR	14.55 ± 6.60	15.92 ± 7.43	−9.4%
	$\epsilon {}_{3D}$	0.19 ± 0.14	0.18 ± 0.12	4.2%
	$\epsilon {}_{3D}^{\prime }$	0.11 ± 0.06	0.11 ± 0.01	0.3%

**Table 9:** Performance scores of AV3T and GAVT on CAV3D. Average TLR [%] and MAE in 2D [pixels] for the AV modality, and summary improvements comparison of GAVT to AV3T ($\Delta_{\mathrm{AV}3T}^{\mathrm{GAVT}}$). The best results across metrics are highlighted with a green background.

		GAVT, AV3T and Comparison (AV), on CAV3D in 2D		
		AV3T	GAVT	$\Delta_{\mathrm{AV}3T}^{\mathrm{GAVT}}$
SOT	TLR	7.00 ± 3.60	13.93 ± 5.41	−99.0%
	$\epsilon {}_{2D}$	16.50 ± 8.60	26.76 ± 10.8	−62.2%
	$\epsilon {}_{2D}^{\prime }$	12.20 ± 0.30	8.69 ± 0.49	28.7%
MOT	TLR	11.20 ± 5.90	21.01 ± 14.7	−87.6%
	$\epsilon {}_{2D}$	24.80 ± 23.7	13.47 ± 10.4	45.7%
	$\epsilon {}_{2D}^{\prime }$	10.1 ± 0.60	9.55 ± 0.18	5.4%
Avg	TLR	9.10 ± 4.75	17.47 ± 10.1	−92.0%
	$\epsilon {}_{2D}$	20.65 ± 16.1	20.11 ± 10.6	2.6%
	$\epsilon {}_{2D}^{\prime }$	11.15 ± 0.45	9.12 ± 0.33	18.2%

**Table 10:** Performance scores of AV3T and GAVT on CAV3D. Average TLR [%] and MAE in 3D [m] for the AV modality, and summary improvements comparison of GAVT to AV3T ($\Delta_{\mathrm{AV}3T}^{\mathrm{GAVT}}$). The best results across metrics are highlighted with a green background.

		GAVT, AV3T and Comparison (AV), on CAV3D in 3D		
		AV3T	GAVT	$\Delta_{\mathrm{AV}3T}^{\mathrm{GAVT}}$
SOT	TLR	31.80 ± 3.50	30.07 ± 4.14	5.4%
	$\epsilon {}_{3D}$	0.30 ± 0.05	0.29 ± 0.04	2.1%
	$\epsilon {}_{3D}^{\prime }$	0.16 ± 0.01	0.13 ± 0.01	21.8%
MOT	TLR	35.70 ± 6.60	32.01 ± 4.13	10.3%
	$\epsilon {}_{3D}$	0.43 ± 0.21	0.35 ± 0.08	18.1%
	$\epsilon {}_{3D}^{\prime }$	0.15 ± 0.01	0.13 ± 0.01	13.7%
Avg	TLR	33.75 ± 5.05	31.04 ± 4.14	8.0%
	$\epsilon {}_{3D}$	0.37 ± 0.13	0.32 ± 0.06	11.5%
	$\epsilon {}_{3D}^{\prime }$	0.16 ± 0.01	0.13 ± 0.01	17.9%

## Data Availability

Publicly available datasets were used in this study. The AV16.3 data can be found at https://www.idiap.ch/en/dataset/av16-3 and the CAV3D data can be requested at https://speechtek.fbk.eu/cav3d-dataset.
